# A parental requirement for dual-specificity phosphatase 6 in zebrafish

**DOI:** 10.1186/s12861-018-0164-6

**Published:** 2018-03-15

**Authors:** Jennifer M. Maurer, Charles G. Sagerström

**Affiliations:** Department of Biochemistry and Molecular Pharmacology, University of Massachusetts Medical School, Worcester, MA USA

**Keywords:** CRISPR, ERK signaling, Dual-specific phosphatase, MAP kinase phosphatase, Germ cell development, Zebrafish embryonic patterning

## Abstract

**Background:**

Signaling cascades, such as the extracellular signal-regulated kinase (ERK) pathway, play vital roles in early vertebrate development. Signals through these pathways are initiated by a growth factor or hormone, are transduced through a kinase cascade, and result in the expression of specific downstream genes that promote cellular proliferation, growth, or differentiation. Tight regulation of these signals is provided by positive or negative modulators at varying levels in the pathway, and is required for proper development and function. Two members of the dual-specificity phosphatase (Dusp) family, *dusp6* and *dusp2*, are believed to be negative regulators of the ERK pathway and are expressed in both embryonic and adult zebrafish, but their specific roles in embryogenesis remain to be fully understood.

**Results:**

Using CRISPR/Cas9 genome editing technology, we generated zebrafish lines harboring germ line deletions in *dusp6* and *dusp2*. We do not detect any overt defects in *dusp2* mutants, but we find that approximately 50% of offspring from homozygous *dusp6* mutants do not proceed through embryonic development. These embryos are fertilized, but are unable to proceed past the first zygotic mitosis and stall at the 1-cell stage for several hours before dying by 10 h post fertilization. We demonstrate that *dusp6* is expressed in gonads of both male and female zebrafish, suggesting that loss of *dusp6* causes defects in germ cell production. Notably, the 50% of homozygous *dusp6* mutants that complete the first cell division appear to progress through embryogenesis normally and give rise to fertile adults.

**Conclusions:**

The fact that offspring of homozygous *dusp6* mutants stall prior to activation of the zygotic genome, suggests that loss of *dusp6* affects gametogenesis and/or parentally-directed early development. Further, since only approximately 50% of homozygous *dusp6* mutants are affected, we postulate that ERK signaling is tightly regulated and that *dusp6* is required to keep ERK signaling within a range that is permissive for proper embryogenesis. Lastly, since *dusp6* is expressed throughout zebrafish embryogenesis, but *dusp6* mutants do not exhibit defects after the first cell division, it is possible that other regulators of the ERK pathway compensate for loss of *dusp6* at later stages.

**Electronic supplementary material:**

The online version of this article (10.1186/s12861-018-0164-6) contains supplementary material, which is available to authorized users.

## Background

The extracellular signal-regulated kinase (ERK) pathway is a major signaling cascade that promotes proliferation and differentiation in many different cell types. As one of the mitogen-activated protein (MAP) kinase pathways, the canonical ERK pathway receives signals from receptors for a growth factor or hormone, such as fibroblast growth factor (FGF), epidermal growth factor (EGF), and platelet-derived growth factor (PDGF), which then activates a MAP kinase kinase kinase (Raf), a MAP kinase kinase (MEK), and finally the MAP kinase ERK. Phosphorylated and activated ERK then moves into the cell nucleus, where it can activate transcription factors to initiate target gene expression. During early development, ERK signaling is active in several critical regions of the zebrafish embryo. For example, ERK signaling works cooperatively with Wnt signaling to promote trunk elongation and the formation of somites in the tailbud [1], and triggers the differentiation of lens fiber cells in the developing eye [2]. It has been demonstrated that ERK signaling is required for proper patterning, especially within the hindbrain, where the cascade is initiated by the FGF pathway, defines the forming rhombomere boundaries, and sets up the anterior-posterior axis [3–6]. Zebrafish embryos treated with an inhibitor of the FGF receptor upstream of ERK lack the fifth and sixth rhombomere (r5 and r6) of the hindbrain and the neurons that normally develop in those regions [5]. Similar to other major signaling pathways, the ERK pathway is able to induce the expression of its own regulators. Many such proteins, including members of the dual-specificity phosphatase (Dusp) and sprouty (Spry) families, Sef, and FLRT, are expressed downstream of the pathway [7]. These proteins interact with upstream pathway components or with ERK itself, and provide positive or negative feedback to modulate the signaling pathway [3, 8–10].

Early embryonic patterning is also driven by the *hox* genes, a key family of homeodomain-containing transcription factors that control cell fate specification [11, 12]. Notably, a microarrary screen identified a Dusp family member, *dusp2* (also called PAC-1 or wu:fj40g04), as a *hoxb1b*-inducible gene in zebrafish [13]. The Dusp family comprises a group of proteins that remove phosphates from both serine/threonine and tyrosine residues of mitogen-activated protein (MAP) kinases resulting in their inactivation. Previous work has shown that Dusp2 is an inducible, nuclear protein that has a strong specificity for ERK [9, 14–19]. There is also evidence that Dusp2 is capable of dephosphorylating p38 in vitro [14] and JNK in vivo [15]. In accordance with it being *hoxb1b-*regulated, *dusp2* is expressed in rhombomere 4 (r4) of the hindbrain – a region that requires *hoxb1b* function. A very similar protein, Dusp6 (also called MKP3), is expressed in several regions of the early embryo, including in r4 where its expression overlaps with *dusp2* and *hoxb1b* [20]. In contrast to Dusp2, Dusp6 is a cytoplasmic protein and has confirmed roles in developmental signaling, including axial patterning, limb development, organ size regulation, and somite formation [20–22]. The fact that *dusp2* and *dusp6* are co-expressed with *hoxb1b* in r4, and that *dusp2* is *hoxb1b* inducible, suggests a potential role for *hox* genes in controlling ERK signaling. Loss of function *dusp2* mice were reported to develop normally, but this was not analyzed in detail [15]. Loss of function *dusp6* mice and morphant zebrafish have been analyzed, and the effects in these animals mimic mutations that inappropriately active FGF receptors [20, 21]. However, these phenotypes differ significantly between the two species. Notably, the analysis in zebrafish made use of anti-sense morpholino oligos (MOs), whose reliability has recently been called into question [23, 24].

Here we used the CRISPR/Cas9 genome editing system to generate loss of function zebrafish mutants for both *dusp2* and *dusp6*. We do not detect any developmental defects in *dusp2* mutants, but find that embryos derived from homozygous *dusp6* mutant parents have reduced viability. These embryos are unable to undergo the first cell division and stall at the 1-cell stage. Our results indicate that this phenotype is independent of the zygotic genome, and instead suggest a parental requirement for *dusp6* in zebrafish embryogenesis.

## Methods

### Zebrafish care

Wildtype Ekkwill and mutant zebrafish lines were raised in the University of Massachusetts Medical School Zebrafish Facility. All embryos were staged according to morphological criteria and hours or minutes post fertilization [25].

### Zebrafish embryonic injections

Embryos were collected from natural matings immediately following fertilization. Collected embryos were aligned on an agarose mold and injected with 1-2 ng of injection mix using a borosil needle, micromanipulator, and dissecting microscope. For the injections of *fgf8* mRNA, a plasmid containing the full coding sequence of *fgf8* was in vitro transcribed. This mRNA was diluted in water and phenol red to a final concentration of 5-500 ng/μl and injected into 1-cell embryos.

### Generation and injection of CRISPR guide RNAs

CRISPR target sites were selected based on their proximity to the start and stop codons of the coding sequence of the targeted gene, and also by the requirement for a protospacer adjacent motif (PAM) sequence (NGG) at the 3′ end of target site. We created and annealed oligos containing a T7 promoter sequence, the target sequence, and an additional constant region to create the template for the guide RNAs (Additional file 1). These templates were transcribed in vitro using T7 RNA polymerase (Promega) in a reaction containing transcription buffer (Promega), RNase inhibitor (Promega), and rNTPs. A linearized plasmid encoding *cas9* [26] was also transcribed in vitro using the Sp6 mMessage mMachine Kit (Ambion). The two guide RNAs targeting each gene were combined with *cas9* mRNA and phenol red, and 1-2 nl of this mixture was injected into the cell of early 1-cell stage embryos.

### Identification of germ line mutations and genotyping

For both *dusp6* and *dusp2* mutants, the embryos injected with the guide RNAs and *cas9* mRNA mixture were raised as the F0 generation. At 3 months of age, these fish were individually crossed to a wildtype fish (Fig. 2c). Half of each resulting clutch was raised to adulthood as the F1 generation. Genomic DNA was extracted from the embryos in the remaining half of the clutch to confirm activity of the guide RNAs. This genomic DNA was screened for deletions by PCR using primers that flank the region between the two guide RNA target sites (Fig. 2a, Additional file 2). Amplification from mutant sequences containing large deletions will produce 400-600 bp products (Fig. 2b, c, Table 1). In contrast, amplification from wildtype sequences will produce products greater than 1 kb and these fragments may not amplify well under the PCR conditions used. F1 adults derived from positive clutches were individually genotyped with fin clip DNA using the same PCR primers. To confirm that these fish were heterozygous, a second set of primers was used to amplify only the wildtype sequence where one or both primers were placed inside the deletion (Fig. 2a, Additional file 2). F1 heterozygous fish were crossed to generate homozygous mutants.

**Table 1:** Characteristics of CRISPR guide RNAs targeting *dusp6* and *dusp2*

CRISPR guide	Target coordinate ^a^	Target sequence ^b^	Strand ^c^	Size of mutant PCR band ^d^	Mutagenesis rate ^e^
dusp6-5′	Chr25:18233489	GAGCCTCATGCTCCGGCGAC	–	~ 564 bp	2/23
dusp6-3′	Chr25:18231243	CTCGAGTCCACGTGAGGTCC	–		
dusp2-5′	Chr8:40589831	GGCGACCCTCTCGAGATCTC	+	~ 392 bp	3/23
dusp2-3′	Chr8:40592681	ACACTGTGACAGATCTACAA	+		

^a^Target coordinate defined by the first nucleotide of the target sequence

^b^Genomic sequence targeted by the guide RNA

^c^Strand of genomic DNA which is targeted by the guide RNA

^d^PCR product size if a CRISPR-induced deletion is present (see Additional file 2 for primer sequences)

^e^The number of F0 germ line positive founders identified out of those screened

### Anti-sense morpholino oligo knockdowns

An anti-sense morpholino oligo (MO) was designed to the *dusp6* translation start site with the sequence 5′-TACCGTGAGACCTTAAAACTGCGGA-3′. A MO targeted to the *dusp2* translation start site with the sequence 5′-GTCGCCGATACCCATGATGCCCTCT-3′ was also designed. As a control, a 5-mismatch control oligo was designed with the sequence 5′-GTCcCCcATAgCCATcATcCCCTCT-3′. All MOs were generated by Gene Tools, LLC and re-suspended in distilled water for a stock solution of 3 mM. The stock solution was further diluted with water and phenol red and 1-2 nl was injected into the yolk of 1-cell stage embryos.

### RNA-seq library preparation

Total RNA was extracted from pools of de-chorionated, de-yolked wildtype and *dusp2*^*um287/um287*^*;dusp6*^*um286/um286*^ embryos at 18hpf using the RNeasy Mini Kit (Qiagen). Three libraries from wildtype embryos and three libraries from *dusp2*^*um287/um287*^*;dusp6*^*um286/um286*^ embryos were then generated from 3μg RNA using the TruSeq Stranded mRNA Library Prep Kit (Illumina). All libraries were analyzed for quality on a bioanalyzer prior to sequencing (Agilent 2100 BioAnalyzer).

### Processing and analysis of RNA-seq data

Fastq files containing strand-specific and filtered reads were processed using the University of Massachusetts Medical School Dolphin web interface [27]. Reads were quality checked using FastQC and aligned to the DanRer7 zebrafish transcriptome using RSEM. After filtering out ribosomal RNA read counts, differentially-expressed genes were identified as those with a greater than 2-fold change in expression between the wildtype and *dusp2*^*um287/um287*^*;dusp6*^*um286/um286*^ samples.

### In situ RNA hybridization, immunostaining, and nuclear staining

For in situ hybridization, embryos were fixed at the appropriate time point in 4% paraformaldehyde and stored in 100% methanol at − 20°C. RNA hybridization was performed as described and was followed by a color reaction using NBT/BCIP or INT/BCIP in 10% polyvinyl alcohol [28]. RNA probes for the following genes were produced by cloning a 900-1000 bp fragment of the coding sequence into a vector and transcribing an anti-sense transcript: *dusp6*, *dusp2*, *krox20*, *hoxb1a*, *six7*, *pea3*, *erm*, *fgf3*, *fgf8*, *valentino*, *bmp2b*, *bmp4*, *chordin*, and *noggin1*. The *otx5* probe was purchased from the Zebrafish International Resource Center.

For whole-mount immunostaining, embryos were fixed in 4% paraformaldehyde/8% sucrose/1× PBS. Fluorescent antibody staining was performed as described previously [29]. Commercially-available primary antibodies used: mouse 3A10 (1:100; Developmental Studies Hybridoma Bank [DSHB]), mouse anti-Islet1/2 (39.4D5; 1:100; DSHB), rabbit anti-phospho-p44/42 MAPK ERK1/2 (1:250; Cell Signaling Technology 4370), rabbit anti-phospho-histone H3 (1:200; Abcam 5176), mouse RMO-44 (1:100; Fisher Scientific 13-0500), and mouse anti-Zn8 (1:1000; DSHB). An antibody against Valentino was generated by immunizing rabbits with a GST-tagged full-length zebrafish Valentino protein. This antibody was purified using an IgG Purification Kit (Dojindo Molecular Technologies) and used at a concentration of 1:100. Secondary antibodies used: goat anti-mouse Alexa Fluor 488 (1:200; Molecular Probes A11001), goat anti-rabbit Alex Fluor 568 (1:200; Molecular Probes A110011), and goat anti-rabbit IgG-HRP (1:1000; Abcam 6789; detected with PerkinElmer’s TSA Plus Fluorescein System).

For nuclear staining, embryos were fixed in 4% paraformaldehyde and stored in 100% methanol at − 20°C. Rehydrated whole embryos were stained with 0.5μg/ml DAPI solution in distilled water for 15 min, and then washed for several hours.

For imaging, embryos older than 24hpf were dissected from the yolk and flat-mounted in 70% glycerol for imaging on bridged coverslips. Images were captured using a Nikon Eclipse E600 microscope equipped with a Nikon 20× Plan Fluor objective and a Zeiss Axiocam 503 color camera. Embryos between 1 and 24hpf were suspended in 3% methyl cellulose for imaging. Images were captured using a Leica M165 FC microscope equipped with a Leica DFC310 FX camera. Embryos younger than 1hpf were mounted in 70% glycerol in depression slides for imaging. All images were imported into Adobe Photoshop and adjustments were limited to contrast, levels, and cropping; all adjustments were applied to the entire image.

### Quantitative PCR

Total RNA was extracted from whole embryos, or from dissected organs of the adult fish, using the RNeasy Mini Kit (Qiagen). At least 100 ng of RNA was used to reverse transcribe cDNA using the High Capacity cDNA Reverse Transcription Kit (Applied Biosystems). The qPCR reaction was carried out using SYBR Green qPCR Master Mix (BioTool) on an Applied Biosystems 7300 PCR System. Results were normalized to those of a housekeeping gene (*b-actin* or *odc1*).

## Results

### Knockdown of *dusp6* and *dusp2* via MO results in a hindbrain phenotype

**Fig. 1:**
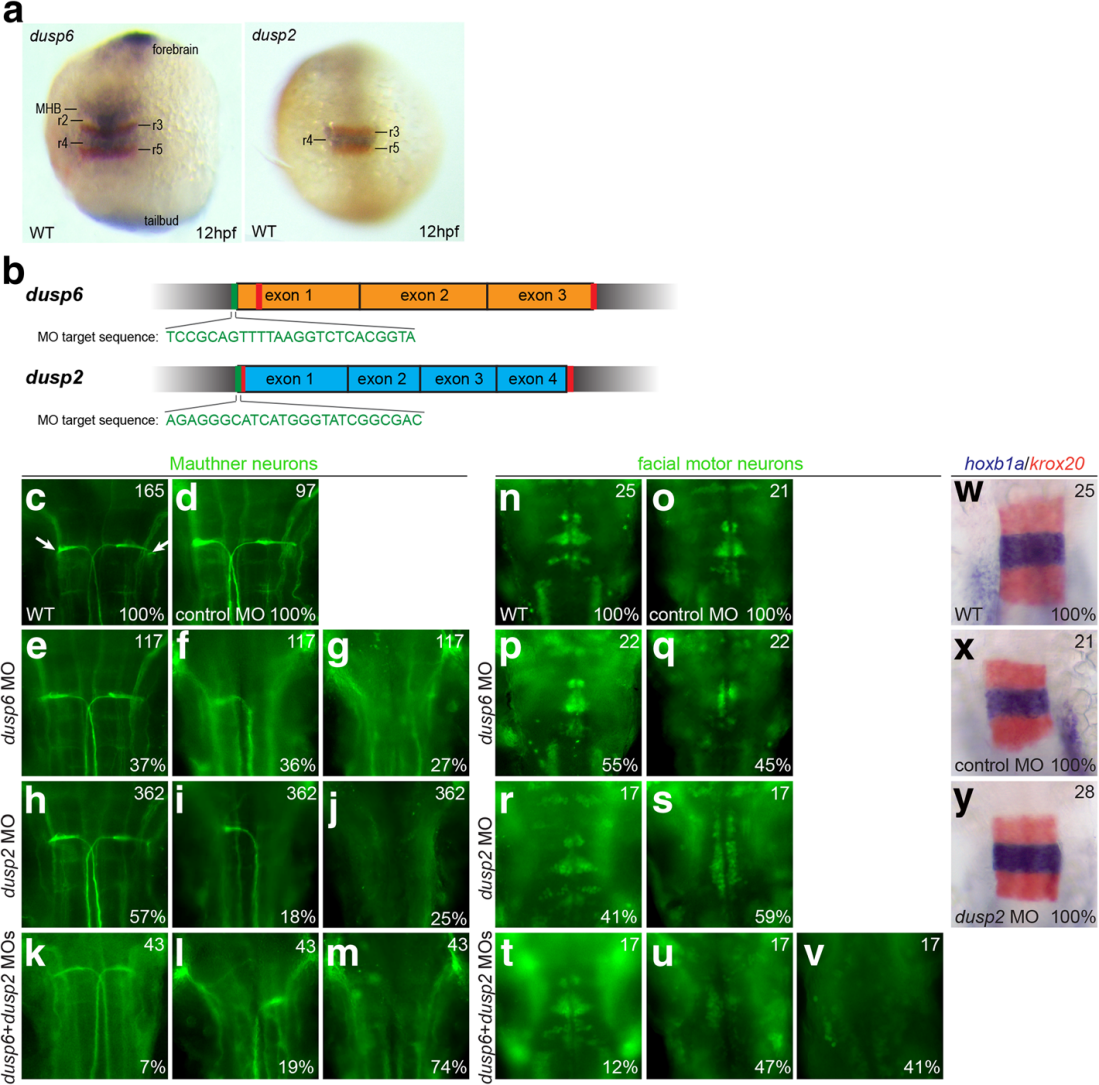
Knockdown of *dusp2* and *dusp6* via MO yields a hindbrain phenotype. **a** 12hpf wildtype embryos were assayed by in situ hybridization for expression of *krox20* (red stain) and *dusp6* (blue stain in left panel) or *dusp2* (blue stain in right panel). **b** Schematic of genomic sequence for *dusp6* and *dusp2*. Red vertical lines indicate CRISPR target sites and green vertical lines indicate MO target sites. **c-v** 48hpf wildtype (**c, n**), control MO-injected (**d, o**), *dusp6* MO-injected (**e-g, p-q**), *dusp2* MO-injected (**h-j, r-s**), and *dusp6* + *dusp2* MO-injected (**k-m, t-v**) embryos were assayed by immunostaining for differentiation of Mauthner neurons (3A10 staining in **c-m**) and facial motor neurons (Islet1/2 staining in **n-v**). **w-y** 18hpf wildtype (**w**), control MO-injected (**x**) and *dusp2* MO-injected (**y**) embryos were assayed by in situ hybridization for expression of *krox20* (red stain) and *hoxb1a* (blue stain). Numbers in top right corner of each panel indicate the total number of embryos assayed for that condition. Numbers in bottom right corner indicate percent of embryos with the phenotype shown. All embryos are in dorsal view with anterior to the top. Embryos in (**a**) are whole-mounts, while embryos in (**c-y**) are flat-mounted and show only the central hindbrain region

We initially used anti-sense morpholino oligos (MOs) to assess the function of *dusp6* and *dusp2* by designing MOs to the translation start site to prevent synthesis of Dusp6 and Dusp2 protein (Fig. 1b). Since *dusp6* and *dusp2* are both expressed in rhombomere 4 (r4) of the hindbrain (Fig. 1a), possibly by acting downstream of *hoxb1b* [13], we examined hindbrain development in MO-injected embryos. r4 is characterized by formation of the Mauthner neurons, a pair of large reticulospinal neurons found in fish and amphibians that are involved in the escape response (Fig. 1c). We find that a large percentage of *dusp6* and *dusp2* morphants are missing one or both Mauthner cells (Fig. 1e-j), while a control MO has no effect (Fig. 1d). Furthermore, injecting a combination of both MOs results in an increase in the occurrence of this phenotype (Fig. 1k-m). Additionally, we notice a minor defect in the patterning of the facial motor neurons that normally migrate from r4 to distinct clusters in the caudal rhombomeres of the hindbrain (Fig. 1n-o). The patterning and clustering of these cells is disrupted in the morphants (Fig. 1p-s). Again, the combination of both MOs results in a more severe phenotype, with some morphants lacking detectable facial motor neurons (Fig. 1t-v). Despite these neuronal defects, the expression of two genes involved in patterning of the early hindbrain appears normal in the morphants (Fig. 1w-y). Additional neurons and markers were examined in the *dusp2* morphants, including the reticulospinal neurons, pERK, *pea3*, *erm*, *fgf8*, *valentino*, and the abducens motor neurons, with no defects (Additional file 3). These results demonstrate that MOs targeting *dusp6* and *dusp2* disrupt the formation and migration of neurons originating in r4 of the hindbrain.

### Generation of *dusp6* and *dusp2* germ line mutants

**Fig. 2:**
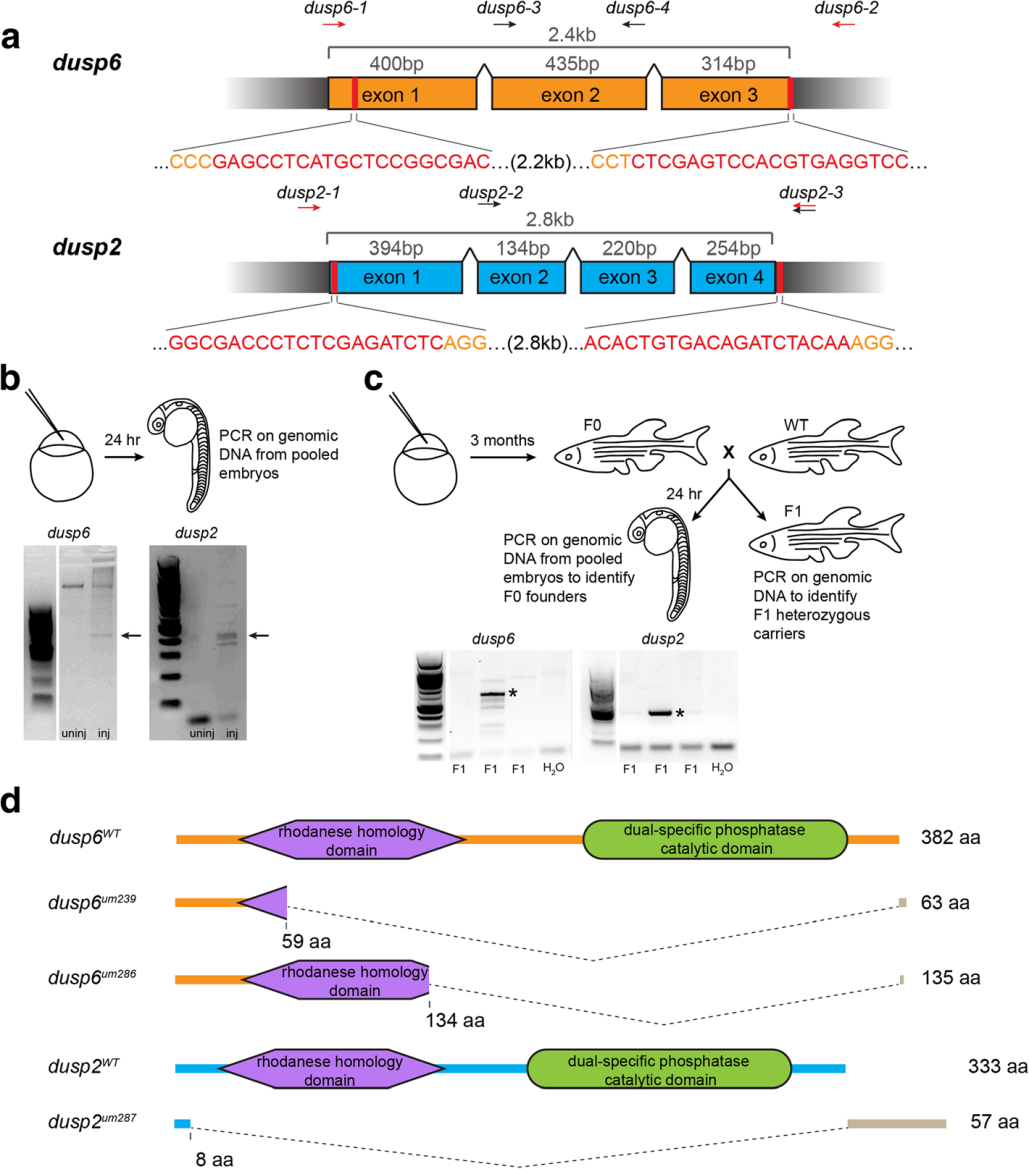
CRISPR genome editing yields loss of function mutants for *dusp6* and *dusp2.* **a** Schematic of the genomic sequence for *dusp6* and *dusp2* with the length of each exon and total coding sequence indicated. Black wedges represent introns. Vertical red lines and red nucleotides denote the CRISPR target sequence, and orange nucleotides indicate PAM sequence. Arrows above each schematic indicate the approximate locations of genotyping primers used to detect CRISPR-induced deletion alleles (red arrows) and wildtype alleles (black arrows; see Additional file 2). **b** Identification of active guide RNAs. Genomic DNA was extracted from pools of injected embryos and PCR-amplified to detect CRISPR-induced deletions. Arrows point to PCR products resulting from successful deletions. **c** Identification of F0 founder fish. Adult F0 fish were crossed to wildtype and the resulting offspring genotyped as in B. Asterisks indicate transmission of deletions to F1 offspring. **d** Predicted peptide sequences of the identified mutant alleles for *dusp6* and *dusp2*. The large dashed wedges represent the location of CRISPR-induced deletions, and the gray bars represent residues that are read out of frame prior to a premature stop codon. Amino acid numbers below each peptide sequence indicate the residue affected by the deletion, and the numbers to the right indicate the length of the resulting peptide

To investigate the roles of *dusp6* and *dusp2* in zebrafish development in greater detail, we set out to generate germ line mutants using the CRISPR/Cas9 genome editing system. We designed two guide RNAs for each gene – one targeted to the 5′ end of the coding sequence and one targeted to the 3′ end (Fig. 2a, Table 1) – with the intention of co-injecting them to delete the sequence between the two target sites. Dusp proteins contain a C-terminal catalytic domain required for substrate recognition and binding [30], as well as an N-terminal rhodanese-homology domain. Although the latter domain is catalytically inactive [31, 32], we nevertheless elected to delete both domains with the goal of generating null alleles. Hence, guide RNA target sequences were chosen based on their proximity to the start and stop codons of the coding sequence, and also by the requirement for a protospacer adjacent motif (PAM) sequence (NGG) at the 3′ end of each target site (Fig. 2a, Table 1).

We injected in vitro transcribed guide RNAs and mRNA encoding *cas9* into early 1-cell stage embryos to test if the guide RNAs were functional. To this end, we prepared genomic DNA from pools of injected embryos at 24hpf and analyzed the target sites by PCR. Using primers that anneal outside the guide RNA target sites (Primers *dusp6-1/dusp6-2* and *dusp2-1/dusp2-3*; Fig. 2a, Additional file 2), we detected bands of approximately 400-600 bp (Fig. 2b), indicating the presence of large deletions created by both the *dusp6* and *dusp2* guide RNA pairs. Each guide RNA pair was then injected into several hundred embryos that were raised to adulthood as the F0 generation (Fig. 2c). This F0 generation is mosaic and each individual fish may carry more than one mutant allele for the same gene. We therefore identified founder fish carrying germ line mutations by crossing F0 individuals to wildtype fish and screening for deletions in the resulting offspring (Fig. 2c) using the same PCR primers (Primers *dusp6-1/dusp6-2* and *dusp2-1/dusp2-3*; Fig. 2a, Additional file 2). F0 founders that were positive for germ line mutations were crossed to wildtype fish and the offspring raised to adulthood followed by genotyping to identify heterozygous F1 carriers.

For *dusp6*, two F0 founders with germ line mutations were identified out of 23 fish tested (Table 1). One founder (*dusp6*^*um239*^) carried a mutant allele with a 2.2 kb deletion within the coding sequence of the *dusp6* gene. The exact nucleotides deleted were determined by sequencing both genomic DNA and cDNA (Additional file 4). This large deletion appears to be the product of two double strand breaks as was expected. Translation of this sequence predicts a 63-amino-acid protein with no known protein domains (Fig. 2d). This founder transmitted this mutation to 13% of its offspring (Table 2). A second founder (*dusp6*^*um286*^) carried a mutant allele with a 1.3 kb deletion spanning exons 2 and 3 of the *dusp6* gene. We suspect that the 5′ guide RNA did not cause a break in this case, and instead the 3′ guide RNA generated a cut that was not properly repaired resulting in a smaller deletion. Translation of the resulting sequence predicts a 135-amino-acid protein that lacks the catalytic domain (Fig. 2d). This founder transmitted this mutation to 24% of its offspring (Table 2).

**Table 2:** Characteristics of *dusp6* and *dusp2* mutant alleles

allele ID	Transmission frequency ^a^	Size of deletion ^b^
*dusp6* ^*um239*^	13.3%	2263 bp
*dusp6* ^*um286*^	23.8%	1308 bp
*dusp2* ^*um287*^	18.2%	2855 bp

^a^Percentage of F1 fish identified as heterozygous carriers of CRISPR-induced deletions

^b^Total number of nucleotides deleted from the genomic sequence

For *dusp2*, three F0 founders with germ line mutations were identified out of 23 fish tested (Table 1). Each of these founders arose from an independent injection, but interestingly, all three carried the same mutant allele containing a 2.8 kb deletion within the coding sequence. Again, the exact nucleotides deleted were determined by sequencing of genomic DNA (Additional file 4). The mutant allele translates to produce a 57-amino-acid protein that lacks any known protein domains (Fig. 2d). The first *dusp2*^*um287*^ founder transmitted this mutation to 18% of its offspring (Table 2). The two additional founders were positive for a deletion by PCR, but we were unable to identify any heterozygous carriers from their offspring. Hence, we have generated two *dusp6* and one *dusp2* alleles that are predicted to lack phosphatase activity.

### *dusp6* and *dusp2* mutants do not recapitulate the morphant phenotype

**Fig. 3:**
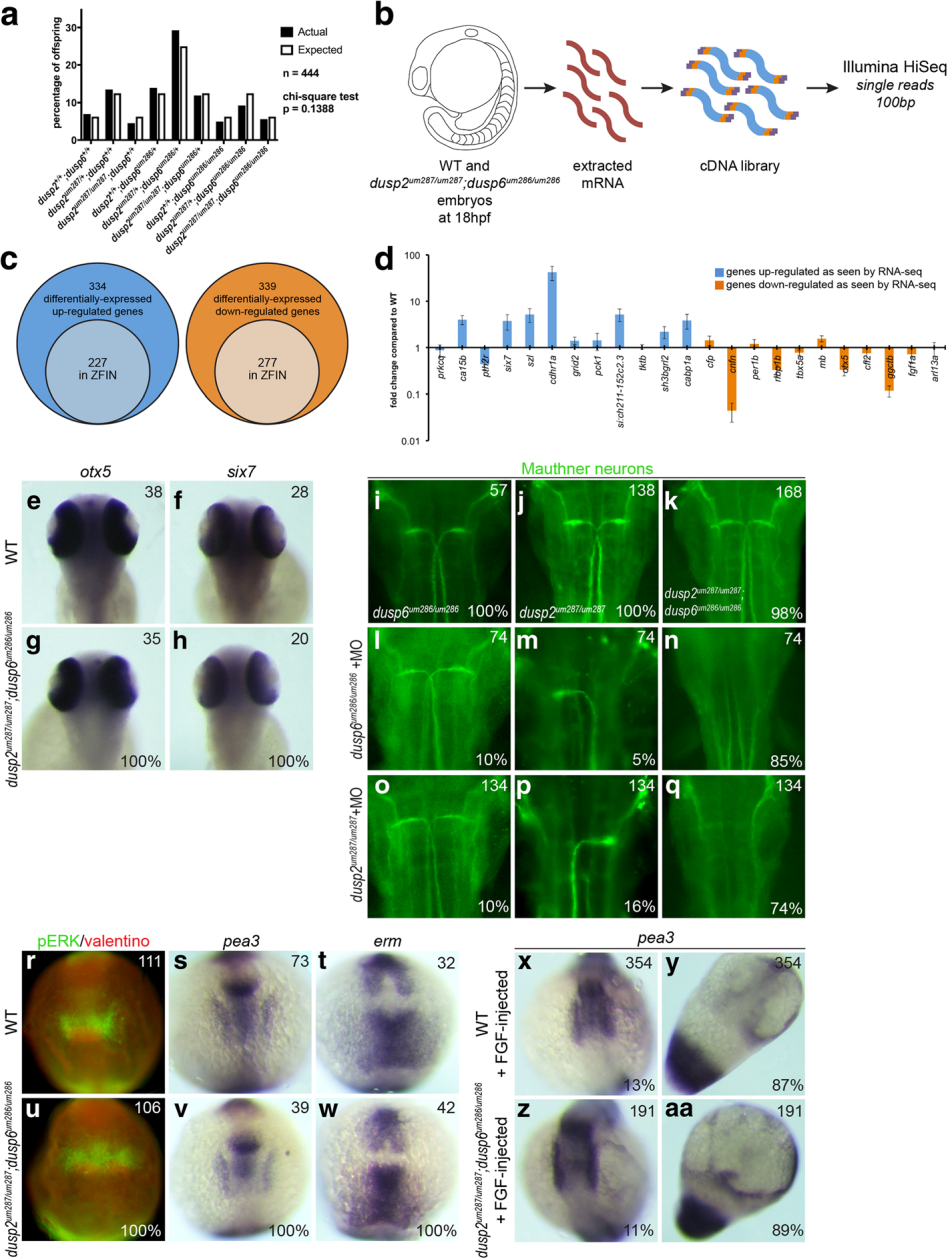
Loss of *dusp6* and *dusp2* does not impact early development. **a** Offspring from crosses between double heterozygous *dusp2*^*um287/+*^*;dusp6*^*um286/+*^ males and females were raised to adulthood and genotyped in order to determine the percentage of each possible genotype in surviving fish. A chi-square test indicates no significant statistical difference between the actual and expected Mendelian ratios. **b** Outline of RNA-seq library production from wildtype and *dusp2/dusp6* double mutant (derived from crosses between *dusp2*^*um287/um287*^*;dusp6*^*um286/um286*^ females and *dusp2*^*um287/um287*^*;dusp6*^*um286/um286*^ males) 18hpf embryos. **c** Diagrams representing the number of differentially expressed up-regulated (left circles) and down-regulated (right circles) genes in *dusp2/dusp6* double mutant (derived from crosses between *dusp2*^*um287/um287*^*;dusp6*^*um286/um286*^ females and *dusp2*^*um287/um287*^*;dusp6*^*um286/um286*^ males) versus wildtype embryos. Inner circles indicate the subset of genes annotated in ZFIN. **d** 23 genes identified as differentially expressed by RNA-seq were re-examined by qPCR on cDNA derived from wildtype versus *dusp2/dusp6* double mutant (derived from crosses between *dusp2*^*um287/um287*^*;dusp6*^*um286/um286*^ females and *dusp2*^*um287/um287*^*;dusp6*^*um286/um286*^ males) embryos. **e-h** 48hpf wildtype (**e, f**) and *dusp2/dusp6* double mutant (derived from crosses between *dusp2*^*um287/um287*^*;dusp6*^*um286/um286*^ females and *dusp2*^*um287/um287*^*;dusp6*^*um286/um286*^ males; **g, h**) embryos were assayed for changes in *otx5* (**e, g**) and *six7* (**f, h**) expression by in situ hybridization. **i-q** Uninjected (**i-k**) or MO-injected (**l-q**) *dusp6* mutant (derived from crosses between *dusp6*^*um286/um286*^ females and *dusp6*^*um286/um286*^ males; **i**), *dusp2* mutant (derived from crosses between *dusp2*^*um287/um287*^ females and *dusp2*^*um287/um287*^ males; **j**), and *dusp2/dusp6* double mutant (derived from crosses between *dusp2*^*um287/um287*^*;dusp6*^*um286/um286*^ females and *dusp2*^*um287/um287*^*;dusp6*^*um286/um286*^ males; **k**) embryos were assayed by immunostaining with 3A10 antibody to detect the Mauthner neurons at 48hpf. **r-w** 12hpf wildtype (**r, s, t**) and *dusp2/dusp6* double mutant (derived from crosses between *dusp2*^*um287/um287*^*;dusp6*^*um286/um286*^ females and *dusp2*^*um287/um287*^*;dusp6*^*um286/um286*^ males; **u, v, w**) embryos were assayed by immunostaining for pERK (green in **r, u**; red counterstain detects the Valentino transcription factor), as well as by in situ hybridization for expression of *pea3* (**s, v**), and *erm* (**t, w**). **x-aa** Wildtype (**x, y**) and *dusp2/dusp6* double mutant (derived from crosses between *dusp2*^*um287/um287*^*;dusp6*^*um286/um286*^ females and *dusp2*^*um287/um287*^*;dusp6*^*um286/um286*^ males; **z, aa**) embryos were injected with *fgf8* mRNA and the expression pattern of *pea3* visualized by in situ hybridization. All embryos are in dorsal view with anterior to the top. Numbers in top right corner of each panel indicate the total number of embryos assayed for that condition. Numbers in bottom right corner indicate percent of embryos with the phenotype shown

While breeding the mutant lines, we found that both *dusp6* and *dusp2* homozygous mutants are viable. Accordingly, crosses between double heterozygous *dusp2*^*um287/+*^*;dusp6*^*um286/+*^ carriers produced offspring with all genotypes represented at the expected ratios and all could be raised to adulthood (Fig. 3a). We took advantage of the homozygous mutant viability and used embryos derived from crosses between double homozygous mutant parents (*dusp2*^*um287/um287*^*;dusp6*^*um286/um286*^ female crossed to *dusp2*^*um287/um287*^*;dusp6*^*um286/um286*^ male) for RNA-seq analysis to identify global changes in gene expression resulting from simultaneous loss of both *dusp6* and *dusp2* (Fig. 3b)*.* Since *dusp6* and *dusp2* are expressed in multiple tissues at segmentation stages, we extracted RNA from pools of 18hpf whole embryos to generate the RNA-seq libraries.

RNA-seq analysis yielded 673 genes that are differentially-expressed between wildtype and mutant embryos out of 23,150 total genes with mapped reads. Of those that are differentially-expressed, 334 are up-regulated and 339 are down-regulated in the mutants (Fig. 3c). We selected 23 differentially expressed genes for validation by quantitative PCR (qPCR) on independently prepared cDNA samples collected from sibling embryos. We find that the expression changes observed by RNA-seq are confirmed by qPCR analysis for 18 of these genes (78%; Fig. 3d).

Next, we narrowed the number of candidate genes down to 504 by pursuing only those with a Zebrafish Information Network (ZFIN, [33]) gene ID number, as these have available information regarding their expression pattern. For this list of differentially-expressed genes, we examined whether there is an enrichment in genes that function within particular pathways, specifically the ERK signaling pathway or another MAPK pathway. Although the PANTHER gene ontology classification system [34–36] grouped 124 of the up- and down-regulated genes into 44 different pathways, there is no clear enrichment for any singular pathway (Additional file 5). We next reasoned that genes expressed in the same regions as *dusp6* and/or *dusp2* would be the best candidates for genes affected in the mutant lines. Using ZFIN’s gene expression database for wildtype fish [37], we analyzed the body structures in which the candidate genes are expressed, with a focus on the regions containing *dusp6* and *dusp2*. Of the 504 genes, 97 are expressed in 25 different structures that overlap with *dusp6* and *dusp2* expression (Additional file 6). We selected two genes (*otx5* and *six7*) that are expressed in the same regions of the forebrain as *dusp6* and *dusp2* and that were also validated by qPCR, but we were unable to detect any change in expression of these genes using in situ hybridization (Fig. 3e-h).

The lack of an apparent phenotype in *dusp* germ line mutants led us to examine if these mutants recapitulate the loss of Mauthner cells observed in *dusp* morphants. Strikingly, Fig. 3i-k shows that Mauthner neurons form normally in *dusp6* mutant embryos (derived from crosses between *dusp6*^*um286/um286*^ females and *dusp6*^*um286/um286*^ males) and in *dusp2* mutant embryos (derived from crosses between *dusp2*^*um287/um287*^ females and *dusp2*^*um287/um287*^ males), as well as in *dusp2/dusp6* double mutants (derived from crosses between *dusp2*^*um287/um287*^*;dusp6*^*um286/um286*^ females and *dusp2*^*um287/um287*^*;dusp6*^*um286/um286*^ males). To examine the cause of this discrepancy further, we injected MOs into the respective mutant line and find that Mauthner cells are lost (Fig. 3l-q). Because the mutant embryos lack the sequences encoding each phosphatase, the loss of Mauthner cells cannot be due to the MOs affecting *dusp* gene expression, but is likely caused by an off-target effect. Since a previous study reported defective *bmp4* and *chordin* expression in *dusp6* morphants [20], we also examined a variety of other genes involved in early embryonic patterning, including *krox20*, *fgf3*, *fgf8*, *bmp2b*, *bmp4*, *chordin*, and *noggin1*, but detect no changes in expression in *dusp2/dusp6* double mutants (derived from crosses between *dusp2*^*um287/um287*^*;dusp6*^*um286/um286*^ females and *dusp2*^*um287/um287*^*;dusp6*^*um286/um286*^ males; Additional file 7). We conclude that zebrafish dorsoventral patterning and Mauthner cell formation is independent of *dusp6* and *dusp2* activity.

To further address the lack of a phenotype, we next investigated the level of pERK (the primary substrate for both Dusp6 and Dusp2) during early segmentation, when both phosphatases are expressed. *dusp2/dusp6* double mutant embryos (derived from crosses between *dusp2*^*um287/um287*^*;dusp6*^*um286/um286*^ females and *dusp2*^*um287/um287*^*;dusp6*^*um286/um286*^ males) stained with an anti-pERK antibody, and counterstained with an anti-Valentino antibody marking r5 and r6 of the hindbrain, show no differences in intensity or location of pERK within the hindbrain or other regions of the embryo compared to wildtype embryos (Fig. 3r, u). We also examined the expression patterns of two ERK target genes, *pea3* and *erm*, of which neither is affected in the mutants (Fig. 3s-t, v-w). Since key signaling pathways, such as the ERK signaling pathway, are held under many levels of regulation [7, 38–42], we considered the possibility that other forms of control could be compensating for the loss of *dusp6* and *dusp2*. Accordingly, we hypothesized that challenging the pathway by exposure to higher levels of ligand might expose a defect in the mutants. To test this, we injected wildtype and *dusp2/dusp6* double mutant (derived from crosses between *dusp2*^*um287/um287*^*;dusp6*^*um286/um286*^ females and *dusp2*^*um287/um287*^*;dusp6*^*um286/um286*^ males) embryos with *fgf8* mRNA, raised them to the early segmentation stages, and then examined the expression pattern of the ERK target gene *pea3*. While excess *fgf8* proved to have a gross effect on early embryonic development and morphology, we did not observe a difference in the effect between wildtype and mutant embryos (Fig. 3x-aa). Hence, despite validated gene expression changes in the mutants, *dusp6* and *dusp2* mutants do not recapitulate the morphant phenotype.

### Homozygous *dusp6* mutant embryos have reduced viability through gastrulation

**Fig. 4:**
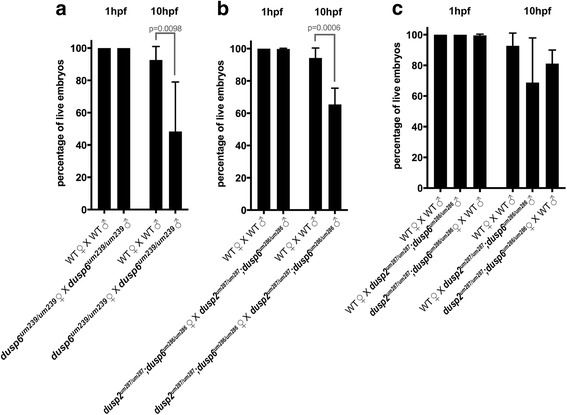
*dusp6* homozygous mutant embryos have reduced viability. **a** Comparison of the percent live embryos at 1hpf and 10hpf between wildtype and *dusp6* mutant (derived from crosses between *dusp6*^*um239/um239*^ females and *dusp6*^*um239/um239*^ males) clutches. Statistical significance was determined by t-test and the *p*-value is indicated. **b** Comparison of the percent live embryos at 1hpf and 10hpf between wildtype and *dusp2/dusp6* double mutant (derived from crosses between *dusp2*^*um287/um287*^*;dusp6*^*um286/um286*^ females and *dusp2*^*um287/um287*^*;dusp6*^*um286/um286*^ males) clutches. Statistical significance was determined by t-test and the p-value is indicated. **c** Comparison of the percent live embryos at 1hpf and 10hpf in clutches derived from wildtype male crossed to wildtype female, wildtype female crossed to *dusp2*^*um287/um287*^*;dusp6*^*um286/um286*^ mutant male, and wildtype male crossed to *dusp2*^*um287/um287*^*;dusp6*^*um286/um286*^ mutant female. ANOVA+Multiple Comparison Analysis did not reveal a statistically significant difference. A minimum of three clutches was analyzed for each cross with the mean percentages displayed ± SD

During our analysis, we noticed that the offspring of *dusp6* homozygous mutant parents has reduced viability during the first 10 h after fertilization. To examine this effect further, wildtype and *dusp6* mutant (derived from crosses between *dusp6*^*um239/um239*^ females and *dusp6*^*um239/um239*^ males) clutches were collected and the number of live embryos counted at 1hpf and 10hpf. We routinely observe that a small percentage (approximately 5%) of wildtype embryos die by the end of gastrulation, but the homozygous *dusp6* mutant embryos show a significant decrease in viability with only approximately 50% of embryos surviving to 10hpf (Fig. 4a). We also examined the viability of *dusp2/dusp6* double mutant embryos (derived from crosses between *dusp2*^*um287/um287*^*;dusp6*^*um286/um286*^ mutant females and *dusp2*^*um287/um287*^*;dusp6*^*um286/um286*^ mutant males). The *dusp2/dusp6* double mutant clutches show a decrease in viability (Fig. 4b) that is indistinguishable from the *dusp6*^*um239/um239*^ mutant clutches (Additional file 8A), indicating that loss of *dusp2* does not decrease viability further and demonstrating that the *dusp6*^*um239*^ and *dusp6*^*um286*^ alleles produce quantitatively similar phenotypes. Lastly, we examined if having one mutant parent is sufficient for reduced viability. To address this, we crossed a wildtype female to a *dusp2*^*um287/um287*^*;dusp6*^*um286/um286*^ mutant male or a wildtype male to a *dusp2*^*um287/um287*^*;dusp6*^*um286/um286*^ mutant female. The survival of embryos from these crosses, while somewhat variable from clutch to clutch, is not statistically different than that of wildtype embryos (Fig. 4c; Additional file 8A), indicating that reduced viability is apparent only when both parents are mutant.

### A fraction of homozygous *dusp6* mutant embryos stall at the first cell division

**Fig. 5:**
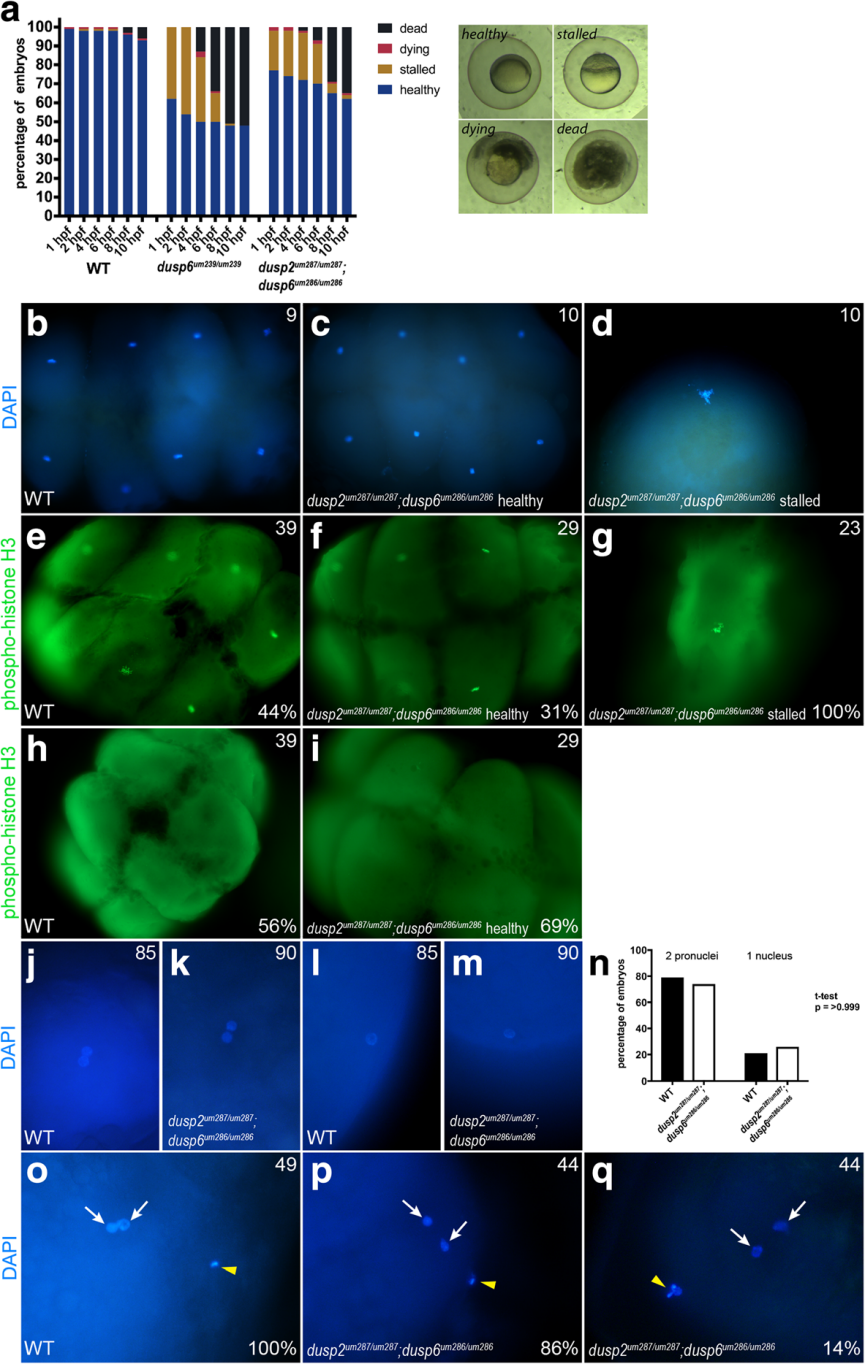
A fraction of *dusp6* homozygous mutant embryos stall at the first cell division. **a** Wildtype, *dusp6* mutant (derived from crosses between *dusp6*^*um239/um239*^ females and *dusp6*^*um239/um239*^ males), and *dusp2/dusp6* double mutant (derived from crosses between *dusp2*^*um287/um287*^*;dusp6*^*um286/um286*^ females and *dusp2*^*um287/um287*^*;dusp6*^*um286/um286*^ males) clutches were monitored throughout early development and the health of each embryo scored at each time point according to the brightfield images to the right (a minimum of three clutches were scored for each cross). Average cumulative counts are shown. **b-d** DAPI staining on 1hpf wildtype and *dusp2/dusp6* double mutant (derived from crosses between *dusp2*^*um287/um287*^*;dusp6*^*um286/um286*^ females and *dusp2*^*um287/um287*^*;dusp6*^*um286/um286*^ males) embryos to visualize the nuclei. **e-i** Immunostaining for phospho-histone H3 to visualize mitotic nuclei of wildtype and *dusp2/dusp6* double mutant (derived from crosses between *dusp2*^*um287/um287*^*;dusp6*^*um286/um286*^ females and *dusp2*^*um287/um287*^*;dusp6*^*um286/um286*^ males) embryos. **j-m** DAPI staining at 10 min post fertilization of wildtype and *dusp2/dusp6* double mutant (derived from crosses between *dusp2*^*um287/um287*^*;dusp6*^*um286/um286*^ females and *dusp2*^*um287/um287*^*;dusp6*^*um286/um286*^ males) embryos to visualize the early pronuclei. **n** Quantification of wildtype and *dusp2/dusp6* double mutant (derived from crosses between *dusp2*^*um287/um287*^*;dusp6*^*um286/um286*^ females and *dusp2*^*um287/um287*^*;dusp6*^*um286/um286*^ males) mutant embryos containing one or two pronuclei. A t-test yields a p-value of > 0.999 indicating no significant statistical difference. **o-q** DAPI staining to detect polar bodies in wildtype and *dusp2/dusp6* double mutant (derived from crosses between *dusp2*^*um287/um287*^*;dusp6*^*um286/um286*^ females and *dusp2*^*um287/um287*^*;dusp6*^*um286/um286*^ males) embryos at 10 min post fertilization. White arrows indicate pronuclei and yellow arrowheads indicate polar bodies in (**o-q**). Numbers in top right corner of each panel indicate the total number of embryos assayed for that condition. Numbers in bottom right corner indicate percent of embryos with the phenotype shown

To further characterize the reduced viability of *dusp6* mutants, we collected clutches of wildtype and homozygous *dusp6* mutant (derived from crosses between *dusp6*^*um239/um239*^ females and *dusp6*^*um239/um239*^ males) embryos and monitored them throughout the cleavage, blastula, and gastrula stages (Fig. 5a). As expected, we again found that 50% of *dusp6* mutant embryos die by 10hpf. Notably, in contrast to wildtype embryos that had all undergone at least one round of cell division by 1hpf, approximately 40-50% of the *dusp6* mutant embryos remained at the 1-cell stage at 1hpf. We refer to these as ‘stalled’ embryos and we monitored their development for the subsequent stages. We find that all of the stalled embryos remain at the 1-cell stage over the next 8 to 10 h until they eventually die. We noticed that some of the stalled embryos proceed to develop a slight cleavage furrow, but they appear unable to complete the process of cell division, and will later return to the smooth cell surface typically seen at the 1-cell stage. When monitoring *dusp2/dusp6* double mutant clutches (derived from crosses between *dusp2*^*um287/um287*^*;dusp6*^*um286/um286*^ females and *dusp2*^*um287/um287*^*;dusp6*^*um286/um286*^ males), we find that they show an identical phenotype to *dusp6*^*um239/um239*^ mutants (Fig. 5a). This is true both in terms of the detailed phenotype (stalling at the 1-cell stage with occasional incipient cleavage furrows), its onset (starting at the 1-cell stage) and duration (all stalled embryos have died by 10hpf), demonstrating that the *dusp6*^*um239*^ and *dusp6*^*um286*^ alleles produce qualitatively indistinguishable phenotypes. We also examined clutches from a wildtype female crossed to a *dusp2*^*um287/um287*^*;dusp6*^*um286/um286*^ mutant male and a wildtype male crossed to a *dusp2*^*um287/um287*^*;dusp6*^*um286/um286*^ mutant female. These exhibited less severe effects (Additional file 8B), further supporting our conclusion that both parents need to be mutant to significantly affect embryo viability.

Although the stalled embryos appear unable to complete cell division, it is unclear if they are progressing through the cell cycle. To address this, we visualized the nuclei of wildtype and *dusp2/dusp6* double mutant (derived from crosses between *dusp2*^*um287/um287*^*;dusp6*^*um286/um286*^ females and *dusp2*^*um287/um287*^*;dusp6*^*um286/um286*^ males) embryos using DAPI at 1hpf. At this time point, wildtype and healthy *dusp2/dusp6* double mutant embryos are entering the 8-cell stage, while stalled *dusp2/dusp6* double mutant embryos remain at the 1-cell stage. Accordingly, wildtype and healthy *dusp2/dusp6* double mutant embryos contain eight DAPI-positive nuclei with varying degrees of condensation likely depending on their position in the cell cycle at the time of fixation (Fig. 5b, c). In contrast, the stalled embryos contain a single large and disorganized DAPI-positive nucleus (Fig. 5d). We conclude that stalled *dusp2/dusp6* double mutant embryos are unable to complete the cell cycle.

To test at what point of the cell cycle the stalled embryos are arresting, we used a phospho-histone H3 antibody to detect mitotic nuclei. Histone H3 becomes phosphorylated at serine 11 during the end of the G2 phase and the early stages of mitosis [43]. Because the first several cell cycles in the developing zebrafish embryo lack G1 and G2 phases [44], positive staining with this antibody should indicate nuclei that are in mitosis and not in interphase. Since the embryos in this experiment derived from natural matings, we expected to see some embryos in mitosis and some in interphase. At 1hpf, when normally developing embryos should enter the 8-cell stage, we find that 44% of wildtype embryos and 31% of healthy *dusp2/dusp6* double mutant embryos (derived from crosses between *dusp2*^*um287/um287*^*;dusp6*^*um286/um286*^ females and *dusp2*^*um287/um287*^*;dusp6*^*um286/um286*^ males) are mitotic, while the remaining embryos are in interphase (Fig. 5e–h-i). In contrast, at 1hpf all stalled *dusp2/dusp6* double mutant embryos contained a single nucleus that is positively stained with the phospho-histone H3 antibody (Fig. 5g). Since the first round of mitosis should have begun at approximately 30 min post fertilization, these embryos must have been stalled in mitosis for at least 30 min prior to fixation. Additionally, all of the stalled embryos contained only one nucleus, as seen by the DAPI and phospho-histone H3 staining, indicating that they do not proceed to anaphase when the sister chromatids are pulled apart. Indeed, separated chromatids are commonly seen in wildtype and healthy *dusp2/dusp6* double mutant embryos at 1hpf (Additional file 9), but are never observed in stalled embryos.

We next examined whether the stalled embryos are fertilized. In the few minutes following fertilization, the maternal and paternal pronuclei condense, migrate towards each other, and merge, allowing the zygote to enter the cell cycle. Hence, the presence of two pronuclei indicates that an embryo has been fertilized. To visualize fertilization, we fixed wildtype and *dusp2/dusp6* double mutant (derived from crosses between *dusp2*^*um287/um287*^*;dusp6*^*um286/um286*^ females and *dusp2*^*um287/um287*^*;dusp6*^*um286/um286*^ males) embryos 10 min post fertilization and stained them with DAPI. Because pronuclear fusion is very rapid and the embryos are collected from natural matings, it is difficult to catch all pronuclei prior to fusion. Accordingly, we find 79% of wildtype embryos contain two detectable pronuclei at 10 min post fertilization, indicating that these embryos are fertilized (Fig. 5j–n). At this early time point, we cannot distinguish between *dusp2/dusp6* double mutant embryos that are healthy and those that will stall at the 1-cell stage. However, if the stalled embryos were not fertilized, we would expect to see an approximate 50% reduction in *dusp2/dusp6* double mutant embryos with two pronuclei, since we know that 50% of them will stall (Fig. 5a). Instead, we find that 74% of *dusp2/dusp6* double mutant embryos contain two detectable pronuclei (Fig. 5k–n), indicating that these embryos are fertilized at the same rate as wildtype embryos. A t-test confirms that there is no significant difference in the fraction of embryos with two pronuclei from wildtype and *dusp2/dusp6* double mutant clutches (Fig. 5n).

DAPI staining at 10 min post fertilization also labels the polar bodies and we noticed that some *dusp2/dusp6* double mutant (derived from crosses between *dusp2*^*um287/um287*^*;dusp6*^*um286/um286*^ females and *dusp2*^*um287/um287*^*;dusp6*^*um286/um286*^ males) embryos have large and disorganized polar bodies (Fig. 5o-q). The frequency of abnormal polar bodies (14%) is lower than the frequency of stalled embryos (approximately 50%) and the polar bodies in *dusp2/dusp6* double mutants do not persist longer than in wildtype embryos (both are degraded by 1hpf), likely ruling out a role for abnormal polar bodies in the stalling of mutant embryos.

We conclude that *dusp2/dusp6* double mutant embryos are fertilized, but approximately 50% of them stall during mitosis of the first embryonic cell division. These embryos remain arrested in the early stages of mitosis for several hours until they die prior to the end of gastrulation.

### *dusp6* is expressed in zebrafish ovaries and testes

**Fig. 6:**
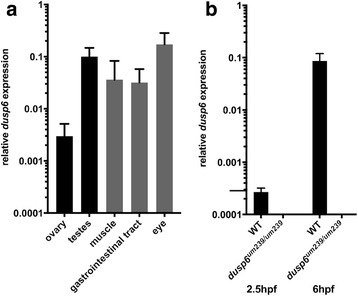
*dusp6* is expressed in ovaries and testes. **a** *dusp6* expression in wildtype adult zebrafish organs was assessed by quantitative RT-PCR using primers *dusp6-3/dusp6-4*. **b** *dusp6* expression in wildtype and *dusp6* mutant (derived from crosses between *dusp6*^*um239/um239*^ females and *dusp6*^*um239/um239*^ males) embryos at 2.5hpf and 6hpf was assessed by quantitative RT-PCR using primers *dusp6-3/dusp6-4*. Results of three independent experiments were normalized to those of *b-actin* and displayed as the mean ± SD

Our analysis revealed that approximately half of *dusp2/dusp6* double mutant embryos stall during the first embryonic cell division. The first several zygotic cell cycles precede activation of the zygotic genome, which occurs at 3-4hpf in zebrafish embryos, and is therefore largely controlled by maternally deposited components supplied during oocyte maturation in the ovary. We therefore determined if *dusp6* transcripts are detectable in the ovary and in the early fertilized embryo. We find that *dusp6* is robustly expressed in the ovary, albeit at somewhat lower levels than in other adult tissues (Fig. 6a). In contrast, *dusp6* is detected at very low levels at maternally controlled stages of embryogenesis (2.5hpf) relative to zygotically controlled stages (6hpf; Fig. 6b), in agreement with a previous report that *dusp6* and *dusp2* transcripts are not maternally deposited in zebrafish [45]. In zebrafish, the large oocytes contribute the majority of cellular volume of the ovary while smaller granulosa cells surround the maturing oocytes and provide growth signals, maternal transcripts, and nutrients via gap junctions. Since *dusp6* is present at very low levels in fertilized oocytes (Fig. 6b), it is likely that *dusp6* is primarily expressed in the granulosa cells, although we cannot exclude the possibility that maternal *dusp6* is specifically degraded in oocytes. Interestingly, *dusp6* is also expressed in the adult testes (Fig. 6a), consistent with our finding that decreased viability is only detected when both parents are homozygous mutants. Based on the current literature [46], it is likely that *dusp6* is expressed in the seminiferous tubules of the testes.

## Discussion

In order to identify a role for *dusp6* and *dusp2* in the developing zebrafish, we generated mutant lines carrying loss of function alleles for these two phosphatases. Our experiments show that ~ 50% of off-spring from homozygous *dusp6* mutant parents stall at the 1-cell stage and die within the first 10 h after fertilization. The affected embryos appear to have been fertilized, but are unable to progress through the first cell cycle. The early onset of the phenotype, coupled with the expression of *dusp6* in both testes and ovaries, lead us to propose a parental role for *dusp6* in permitting zebrafish development to progress past the 1-cell stage. In contrast, loss of *dusp2* function does not affect embryo viability and we have been unable to identify a role for *dusp2* in zebrafish embryogenesis.

### *dusp6* may act to maintain ERK signaling within a permissive range

A key observation regarding the stalling of homozygous *dusp6* mutant embryos is that only a portion of each clutch is affected (approximately 50%, Fig. 4a, b, Fig. 5a), demonstrating that the phenotype is incompletely penetrant. This implies that not all oocytes and/or sperm produced by homozygous mutant adults are defective, but the basis of the incomplete penetrance is unclear. Differences in expression and concentration of signaling components is known to contribute to variability in signaling intensity among individual cells [47, 48], and studies of various signaling pathways have identified roles for redundant regulators in reducing signal noise [49, 50]. Specifically, there is evidence of cell-to-cell variability in levels of protein kinase signaling, and negative feedback regulators such as Dusp proteins are thought to act to minimize such variations [51]. We therefore hypothesize that *dusp6* is required to minimize variations in ERK signaling during gametogenesis and that when *dusp6* is lost, a fraction of oocytes and spermatocytes become exposed to ERK signaling outside the permissive range. Under this model, many oocytes and spermatocytes in the mutants would still be exposed to levels of ERK signaling that fall within the permissive range, but a percentage would receive excessive ERK signals leading to abnormal gametogenesis. In support of such a model, while the phenotype of *Dusp6* mutant mice is distinct from that in zebrafish (see below), it is nevertheless incompletely penetrant [21], consistent with a more general role for *dusp6* in maintaining a permissive range of ERK activity.

However, the possibility remains that the incomplete penetrance results from other causes, such as variations in genetic background, epigenetic factors, individual variations in expressivity, or partial compensation from other regulators. In particular, multiple studies have noted that *dusp6* is expressed in many of the same regions of the zebrafish as FGF ligands [7, 10, 41, 52, 53]. Several other proteins known to regulate FGF signaling are also expressed in these regions, and for this reason they have been referred to as the FGF-synexpression group. This group includes other Dusp proteins and phosphatases, members of the Spry family, Sef, and Flrt [7, 41, 52, 53]. Since these proteins are present in the same regions and modulate the same pathway, it is possible that they compensate for each other when necessary. To address possible compensation, we analyzed the list of differentially-expressed genes from our RNA-seq experiment to see if other negative ERK modulators of the FGF-synexpression group were up-regulated in *dusp6* and *dusp2* mutants. We did not detect significant changes in expression level of any of these genes, but it remains possible that factors regulated by post-translational modifications could compensate for the loss of Dusp function.

### Parental *dusp6* activity is required for zebrafish development to progress past the 1-cell stage

We note that the mutant phenotype is observed very early – shortly after fertilization – and only in off-spring derived from two homozygous mutant parents. This indicates that the phenotype is independent of the zygotic genome and suggests that *dusp6* activity may be required either in the fertilized zygote (following parental deposition into the gametes), or during gametogenesis in the gonads. We tend not to favor the first possibility since we detect only very low levels of *dusp6* in oocytes and sperm are thought not to contain much mRNA, but we cannot exclude the possibility that Dusp6 protein is deposited in oocytes and sperm. In support of the latter model, we find that zebrafish *dusp6* is expressed in both ovaries and testes and work in other systems has shown that ERK signaling is essential for gametogenesis. In particular, luteinizing hormone (LH) and follicle stimulating hormone (FSH) are the primary drivers of ovarian follicle growth and stimulators of the granulosa cells surrounding the developing oocyte [54]. A study performed on rat granulosa cells demonstrated that Dusp6 in the granulosa cells keeps ERK inactivated in the absence of FSH [55]. Once maturation is initiated by FSH, PKA is activated through cAMP to inhibit Dusp6, thereby allowing active pERK to accumulate and drive downstream genes promoting oocyte maturation and progression through the meiotic cell cycle [56]. Other genes activated by ERK, such as *has2* and *ptgs2*, are required for the expansion and growth of the granulosa cells within the ovarian follicle [57, 58]. Similarly, spermatogenesis also requires well-coordinated ERK signaling. Cell cycle regulators and condensation factors downstream of ERK are required for proper chromatid separation and condensation maintenance between rounds of meiosis [59–61]. Similar to the granulosa cells of the ovary, Sertoli cells coordinate meiotic progression of the developing spermatocytes and their growth within the testes [62]. Genes downstream of ERK also ensure the integrity of vital tight junctions between the Sertoli cells and spermatocytes during maturation [63]. Hence, zebrafish *dusp6* mutants may suffer from excess ERK signaling in these pathways that could in turn affect the activity of downstream targets such as cell cycle regulators. Accordingly, there is evidence that mis-regulation of ERK signaling within the mammalian reproductive system results in abnormal pubertal development and infertility. Similarly, female mice carrying a mutant allele for constitutively active RAS have defects in ovulation, and ERK1/2 mutant female mice are completely infertile [64, 65]. Additionally, congenital hypogonadotropic hypogonadism in humans affects both males and females and has been linked to missense mutations in *DUSP6* and other ERK regulators [66].

We note that even if the primary defect in *dusp6* mutants occurs during gametogenesis, it does not become manifest until after fertilization. Furthermore, since the phenotype is most pronounced when both parents are mutant, it suggests that an ERK-dependent event requiring both maternal and paternal input is likely affected in the mutant embryos. A plausible candidate for such a process is the formation of embryonic centrosomes, which requires centrioles provided by the sperm, but also centrosomal components stored in the oocyte [67]. In this scenario, off-spring from two mutant parents would suffer defects in both maternal and paternal centrosomal components and have more severe phenotypes than off-spring derived from only one mutant parent. Indeed, previous work indicates that ERK signaling is required for centrosome duplication [68, 69] and function [70], at least in cell culture models. Hence, *dusp6* activity may be required to keep ERK signaling in a permissive range during gametogenesis and failure to do so may produce defective gametes unable to support the first cell division, possibly due to abnormal centrosome function.

### *dusp6* mutants reveal species-specific defects

Previous work revealed that *Dusp6* mutant mice exhibit increased pERK and *Erm* expression, skeletal dwarfism, craniosynostosis, and hearing loss [21]. All of these defects share characteristics with FGF receptor activating mutations, consistent with a role for *Dusp6* as a negative regulator of ERK signaling. Similar to *dusp6* mutant zebrafish, *Dusp6* mutant mice have increased postnatal lethality, with a significant decrease in homozygous mutant pups surviving to weaning age. However, the mouse mutants die at later stages than the zebrafish mutants, suggesting that these phenotypes are somewhat distinct. Also, while previous published analyses of *dusp6* morphants demonstrated dorsalization and expansion of neural domains [20] and we initially observed defects in *dusp6* morphants, none of these phenotypes are recapitulated in *dusp6* germ line mutants. Several recent publications have found similar instances where germ line mutants do not have the same phenotype as the corresponding morphant [23, 24] and, whereas there may be several causes for such discrepancies, our finding that *dusp6* MOs produce a phenotype in *dusp6* mutants suggests that in our case the morphant phenotype is due to a morpholino off-target effect. Although these analyses suggest that *dusp6* may have species-specific roles, these roles nevertheless appear closely related to ERK signaling in each case. Indeed, it is possible that the observed species differences are due to variable compensation by other ERK-signaling components in different species.

## Conclusions

Our results presented here suggest a parental role for *dusp6* in controlling progression through the first cell division in zebrafish. Tight regulation of ERK signaling is vital for these processes and a loss of function *dusp6* allele may result in a shift of active ERK levels. While some gametes develop under permissive conditions in the mutants, others may be exposed to elevated ERK levels and this may negatively impact their maturation. The embryos resulting from the union of a defective egg and defective sperm stall at the 1-cell stage, unable to complete the first mitosis, and die by 10hpf. However, homozygous mutant embryos from unaffected gametes develop with no overt phenotypes, suggesting that other ERK regulators are able to compensate during embryonic development.

## Additional files

Additional file 1: Sequences of oligos to generate CRISPR guide RNAs. The sequence of the genomic target, the PAM sequence, the sequences of the oligos used as a template are shown for each guide RNA. (DOCX 11 kb)

Additional file 2: Primer sequences to genotype mutants. For both *dusp6* and *dusp2*, two sets of PCR primers were used to genotype: one set to amplify the deletion allele and one set to amplify the wildtype allele. (DOCX 13 kb)

Additional file 3: Additional neuronal and patterning markers examined in *dusp2* morphants. Wildtype, control MO-injected, and *dusp2* MO-injected embryos were analyzed by in situ hybridization for the expression of *pea3*, *erm*, *fgf8*, and *valentino* and by immunostaining to visualize the reticulospinal neurons, pERK, and the abducens motor neurons. (TIFF 3043 kb)

Additional file 4: Nucleotide and predicted amino acid sequence of mutant alleles. A, B. Nucleotide and predicted amino acid sequence of *dusp6* (A) and *dusp2* (B) mutant alleles. Genomic DNA was amplified using primer pairs dusp6-1/dusp6-2 and dusp2-1/dusp2-3 (See Fig. 2a and Additional file 2). Uppercase nucleotides indicate the coding sequence and lowercase nucleotides represent the UTR. Green and red nucleotides indicate translation start and stop sites, respectively. Ellipses (...) indicate the continuation of wild type sequences. Dashes (--) indicate deletions and blue nucleotides indicate insertions in the mutant alleles. Predicted protein sequences are indicated in one letter IUPAC code with purple highlighting the rhodanese homology domain and green highlighting the catalytic domain. Asterisks denote the stop codon. Orange residues are predicted to be encoded by the mutant alleles before encountering a stop codon. C. Transcripts are produced in *dusp6* mutant embryos. cDNA was amplified from homozygous mutant *dusp6* embryos by end-point PCR using the same primers as in A. Sequencing of these transcripts identified the same lesions detected in genomic DNA in A. Dashed lines indicate where gel was cut. (TIFF 1730 kb)

Additional file 5: Gene ontology grouping of differentially-expressed genes. Representation of gene ontology grouping of 124 differentially-expressed genes into 44 different signaling pathways performed by Panther. (TIFF 1233 kb)

Additional file 6: Differentially-expressed genes in the same body structures as *dusp6* and *dusp2*. The left column contains a list of all body structures of the zebrafish in which *dusp6* and/or *dusp2* are expressed, and the right column contains the identified differentially-expressed genes also expressed in those structures. (DOCX 14 kb)

Additional file 7: Additional patterning markers examined in *dusp* mutants. Wildtype (A, C, E, G, I, K, M, O, Q), *dusp2/dusp6* double mutant (derived from crosses between *dusp2*^*um287/um287*^*;dusp6*^*um286/um286*^ females and *dusp2*^*um287/um287*^*;dusp6*^*um286/um286*^ males; B, D, F, H, J, L, N), *dusp2* mutant (derived from crosses between *dusp2*^*um287/um287*^ females and *dusp2*^*um287/um287*^ males; P) and *dusp6* mutant (derived from crosses between *dusp6*^*um239/um239*^ females and *dusp6*^*um239/um239*^ males; R) embryos were analyzed by in situ hybridization for the expression of *krox20*, *fgf3*, *fgf8*, *bmp2b*, *bmp4*, *chordin*, and *noggin1*. (TIFF 3617 kb)

Additional file 8: Offspring from a single mutant parent have a milder phenotype. A. Comparison of the percent live embryos at 1hpf and 10hpf in clutches derived from various crosses between wildtype and homozygous mutant fish (specific genotypes are indicated below each bar). ANOVA+Dunnett’s Multiple Comparison Test revealed a statistically significant decrease in live embryos at 10 hpf for clutches where both parents are homozygous mutant for dusp6, but not when only one parent is homozygous mutant. A minimum of three clutches was analyzed for each cross with the mean percentages displayed ± SD. B. Comparison of the percent live embryos from 1hpf to10hpf in clutches derived from crosses between wildtype and homozygous mutant fish (specific genotypes are indicated below the graph). A minimum of three clutches was analyzed for each cross with the mean percentages displayed ± SD. (TIFF 2486 kb)

Additional file 9: DAPI staining detects embryos undergoing mitosis. Nuclear staining at approximately 1hpf shows that both wildtype and healthy *dusp2/dusp6* double mutant (derived from crosses between *dusp2*^*um287/um287*^*;dusp6*^*um286/um286*^ females and *dusp2*^*um287/um287*^*;dusp6*^*um286/um286*^ males) embryos can be detected at stages of mitosis when the chromatids are separated. (TIFF 841 kb)

## Abbreviations

bp: Basepair
cAMP: Cyclic adenosine monophosphate
CRISPR: Clustered Regularly Interspaced Short Palindromic Repeats
DAPI: 4′,6-diamidino-2-phenylindole
Dusp: Dual specificity phosphatase
ERK: Extracellular signaling regulated kinase
Fgf: Fibroblast growth factor
FSH: Follicle stimulating hormone
hpf: Hours post fertilization
kb: Kilo basepair
LH: Luteinizing hormone
MAP: Mitogen activated protein
MEK: MAP/ERK kinase
MO: Morpholino oligonucleotide
NTP: Nucleoside tri-phosphate
PAM: Protospacer adjacent motif
PKA: Protein kinase A
qPCR: Quantitative polymerase chain reaction
r: Rhombomere
Spry: Sprout
