# Supplementary material for: A parental requirement for dual-specificity phosphatase 6 in zebrafish
Source: BMC Dev Biol. 2018 Mar 15;18:6. doi: 10.1186/s12861-018-0164-6 (PMC5856328; doi:10.1186/s12861-018-0164-6)
Supplement: Supplementary file 1 — Sequences of oligos to generate CRISPR guide RNAs. The sequence of the genomic target, the PAM sequence, the sequences of the oligos used as a template are shown for each guide RNA. (DOCX 11 kb) [file 12861_2018_164_MOESM1_ESM.docx]

**Additional File 1. Sequences of oligos to generate CRISPR guide RNAs**

| **CRISPR** | **Target sequence ^a^** | **PAM ^b^** | **First oligo ^c^** | **Second oligo ^d^** |
| --- | --- | --- | --- | --- |
| dusp6-5′ | GAGCCTCATGCTCCGGCGAC | GGG | TTAATACGACTCACTATAGGTCGCCGGAGCATGAGGCTCGGGGTTTTAGAGCTAGAAATAGCAAG | AAAAAAGCACCGACTCGGTGCCACTTTTTCAAGTTGATAACGGACTAGCCTTATTTTAACTTGCTATTTCTAGCTCTAAAAC |
| dusp6-3′ | CTCGAGTCCACGTGAGGTCC | AGG | TTAATACGACTCACTATAGGACCTCACGTGGACTCGAGAGGGTTTTAGAGCTAGAAATAGCAAG | AAAAAAGCACCGACTCGGTGCCACTTTTTCAAGTTGATAACGGACTAGCCTTATTTTAACTTGCTATTTCTAGCTCTAAAAC |
| dusp2-5′ | GGCGACCCTCTCGAGATCTC | AGG | TTAATACGACTCACTATAGGCGACCCTCTCGAGATCTCAGGGTTTTAGAGCTAGAAATAGCAAG | AAAAAAGCACCGACTCGGTGCCACTTTTTCAAGTTGATAACGGACTAGCCTTATTTTAACTTGCTATTTCTAGCTCTAAAAC |
| dusp2-3′ | ACACTGTGACAGATCTACAA | AGG | TTAATACGACTCACTATAGACACTGTGACAGATCTACAAAGGGTTTTAGAGCTAGAAATAGCAAG | AAAAAAGCACCGACTCGGTGCCACTTTTTCAAGTTGATAACGGACTAGCCTTATTTTAACTTGCTATTTCTAGCTCTAAAAC |

^a^ Genomic sequence targeted by the guide RNA

^b^ Protospacer adjacent motif (PAM) sequence in the genomic DNA recognized by Cas9

^c^ Sequence of the first oligo used to create the guide template containing the T7 promoter sequence

^d^ Sequence of the second oligo used to create the guide template containing the constant region
