# Supplementary material for: A parental requirement for dual-specificity phosphatase 6 in zebrafish
Source: BMC Dev Biol. 2018 Mar 15;18:6. doi: 10.1186/s12861-018-0164-6 (PMC5856328; doi:10.1186/s12861-018-0164-6)
Supplement: Supplementary file 2 — Primer sequences to genotype mutants. For both dusp6 and dusp2, two sets of PCR primers were used to genotype: one set to amplify the deletion allele and one set to amplify the wildtype allele. (DOCX 13 kb) [file 12861_2018_164_MOESM2_ESM.docx]

**Additional File 2. Primers used to amplify wildtype and mutant *dusp6* and *dusp2***

| **Primer name** | **Sequence** | **Purpose** |
| --- | --- | --- |
| *dusp6-1* | CGGTAGAGTGGCTGAAGGAG | Forward primer to amplify wild type and mutant *dusp6* |
| *dusp6-2* | TCCCAAAAACAGGCAAGTCT | Reverse primer used with primer *dusp6-1* |
| *dusp6-3* | GTTCCTCAAGCAGCAGTTCC | Forward primer to amplify only wild type *dusp6* |
| *dusp6-3* | AGAGGTTCTGGCTCCAGTGA | Reverse primer used with primer *dusp6-3* |
| *dusp2-1* | GGAACAATATTGATTTGTGTCACC | Forward primer to amplify wild type and mutant *dusp2* |
| *dusp2-2* | CTTTCTTTTCCTGGGCAGTG | Forward primer to amplify only wild type *dusp2* |
| *dusp2-3* | GTAGAGGTTCGGGGACACG | Reverse primer used with primers *dusp2-1* or *dusp2-2* |
