# Supplementary figures and images for: A parental requirement for dual-specificity phosphatase 6 in zebrafish
Source: BMC Dev Biol. 2018 Mar 15;18:6. doi: 10.1186/s12861-018-0164-6 (PMC5856328; doi:10.1186/s12861-018-0164-6)

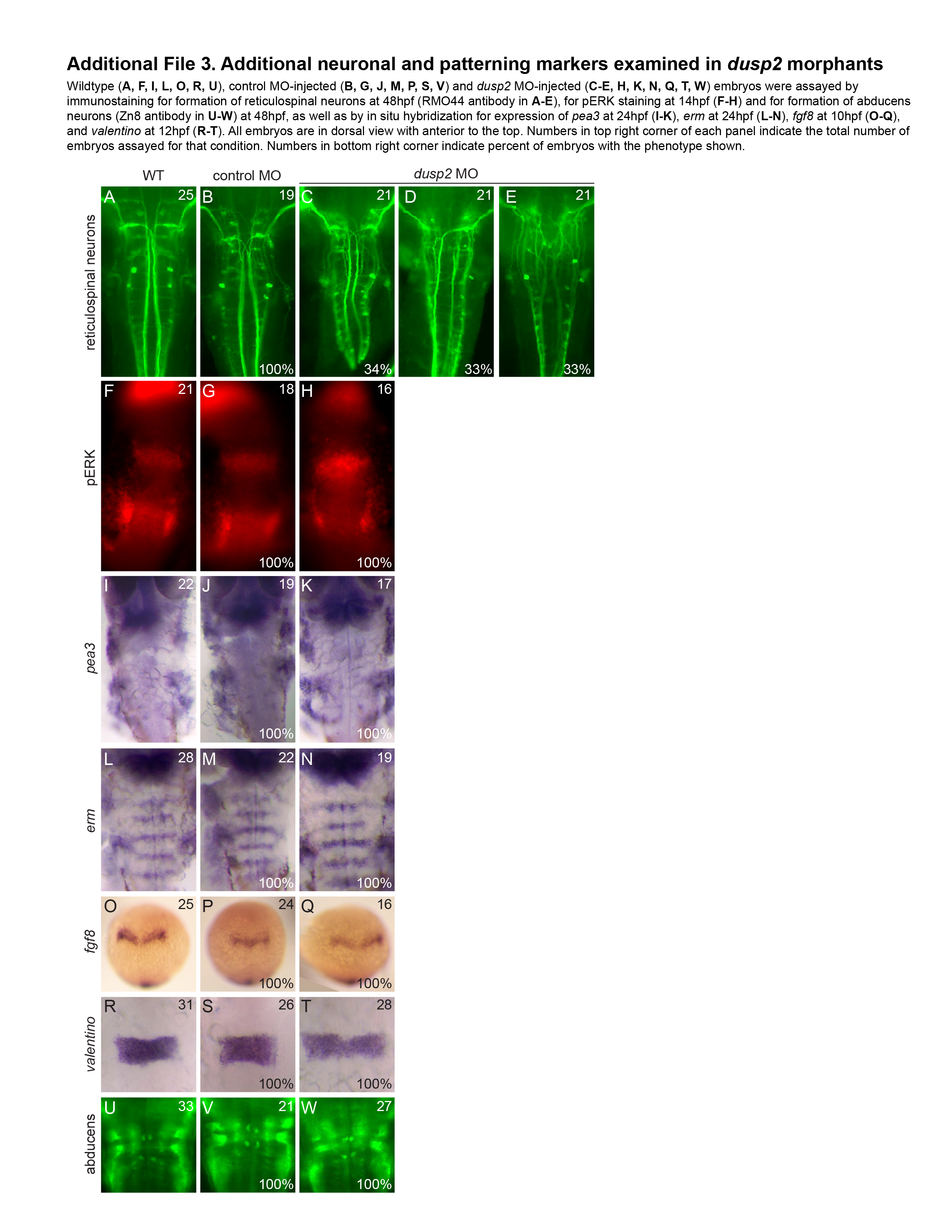

Supplement: Supplementary file 3 — Additional neuronal and patterning markers examined in dusp2 morphants. Wildtype, control MO-injected, and dusp2 MO-injected embryos were analyzed by in situ hybridization for the expression of pea3, erm, fgf8, and valentino and by immunostaining to visualize the reticulospinal neurons, pERK, and the abducens motor neurons. (TIFF 3043 kb) [file 12861_2018_164_MOESM3_ESM.tif]

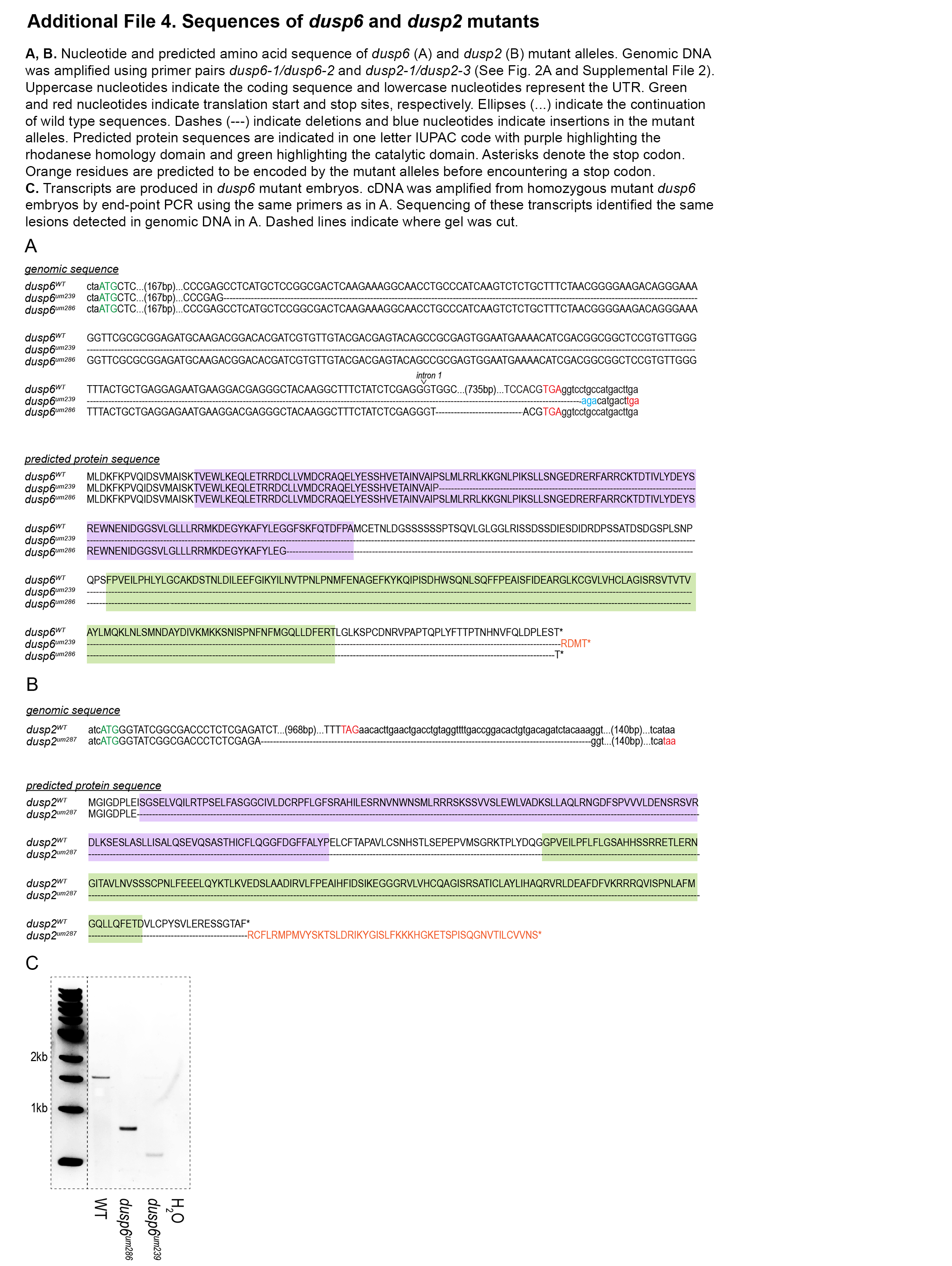

Supplement: Supplementary file 4 — Nucleotide and predicted amino acid sequence of mutant alleles. A, B. Nucleotide and predicted amino acid sequence of dusp6 (A) and dusp2 (B) mutant alleles. Genomic DNA was amplified using primer pairs dusp6-1/dusp6-2 and dusp2-1/dusp2-3 (See Fig. 2a and Additional file 2). Uppercase nucleotides indicate the coding sequence and lowercase nucleotides represent the UTR. Green and red nucleotides indicate translation start and stop sites, respectively. Ellipses (...) indicate the continuation of wild type sequences. Dashes (--) indicate deletions and blue nucleotides indicate insertions in the mutant alleles. Predicted protein sequences are indicated in one letter IUPAC code with purple highlighting the rhodanese homology domain and green highlighting the catalytic domain. Asterisks denote the stop codon. Orange residues are predicted to be encoded by the mutant alleles before encountering a stop codon. C. Transcripts are produced in dusp6 mutant embryos. cDNA was amplified from homozygous mutant dusp6 embryos by end-point PCR using the same primers as in A. Sequencing of these transcripts identified the same lesions detected in genomic DNA in A. Dashed lines indicate where gel was cut. (TIFF 1730 kb) [file 12861_2018_164_MOESM4_ESM.tif]

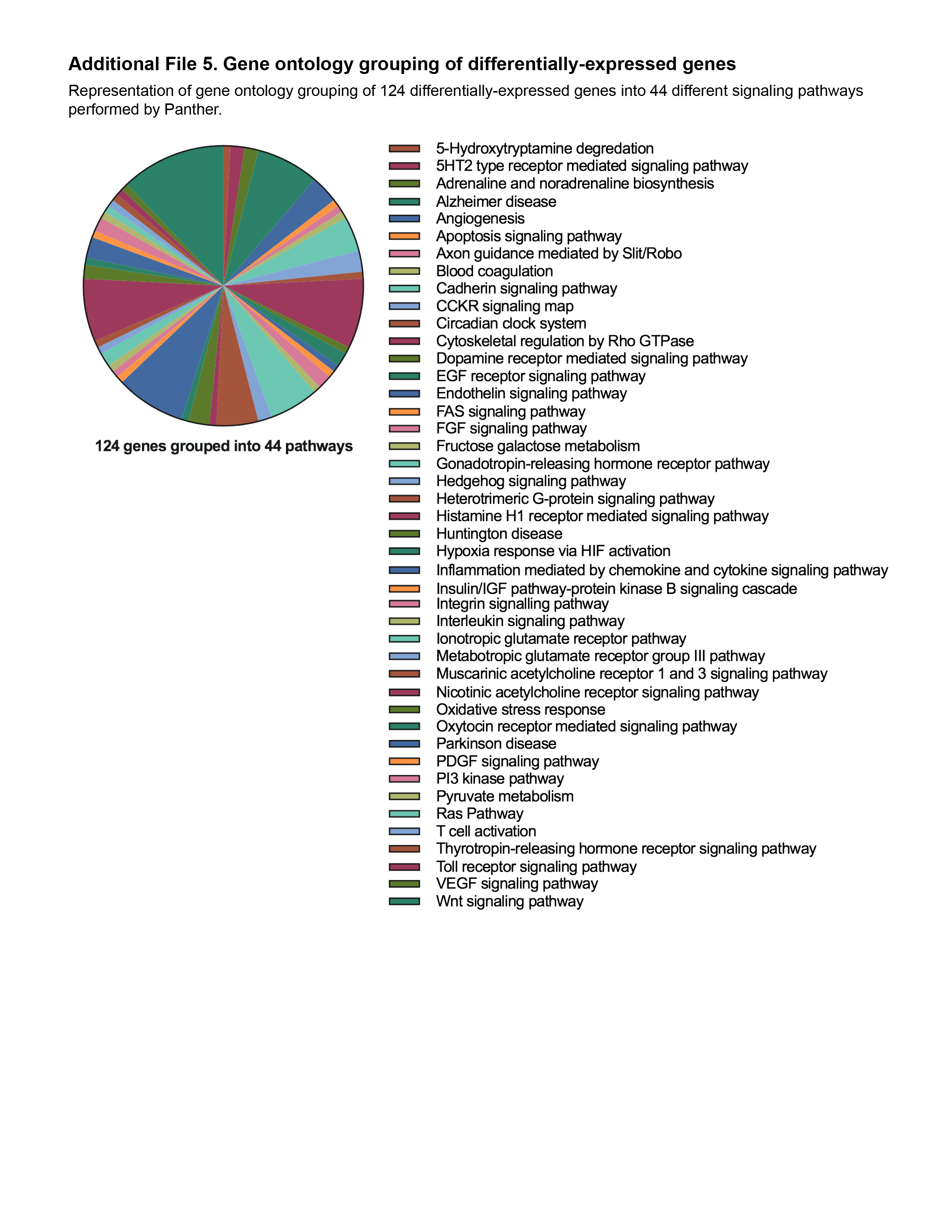

Supplement: Supplementary file 5 — Gene ontology grouping of differentially-expressed genes. Representation of gene ontology grouping of 124 differentially-expressed genes into 44 different signaling pathways performed by Panther. (TIFF 1233 kb) [file 12861_2018_164_MOESM5_ESM.tif]

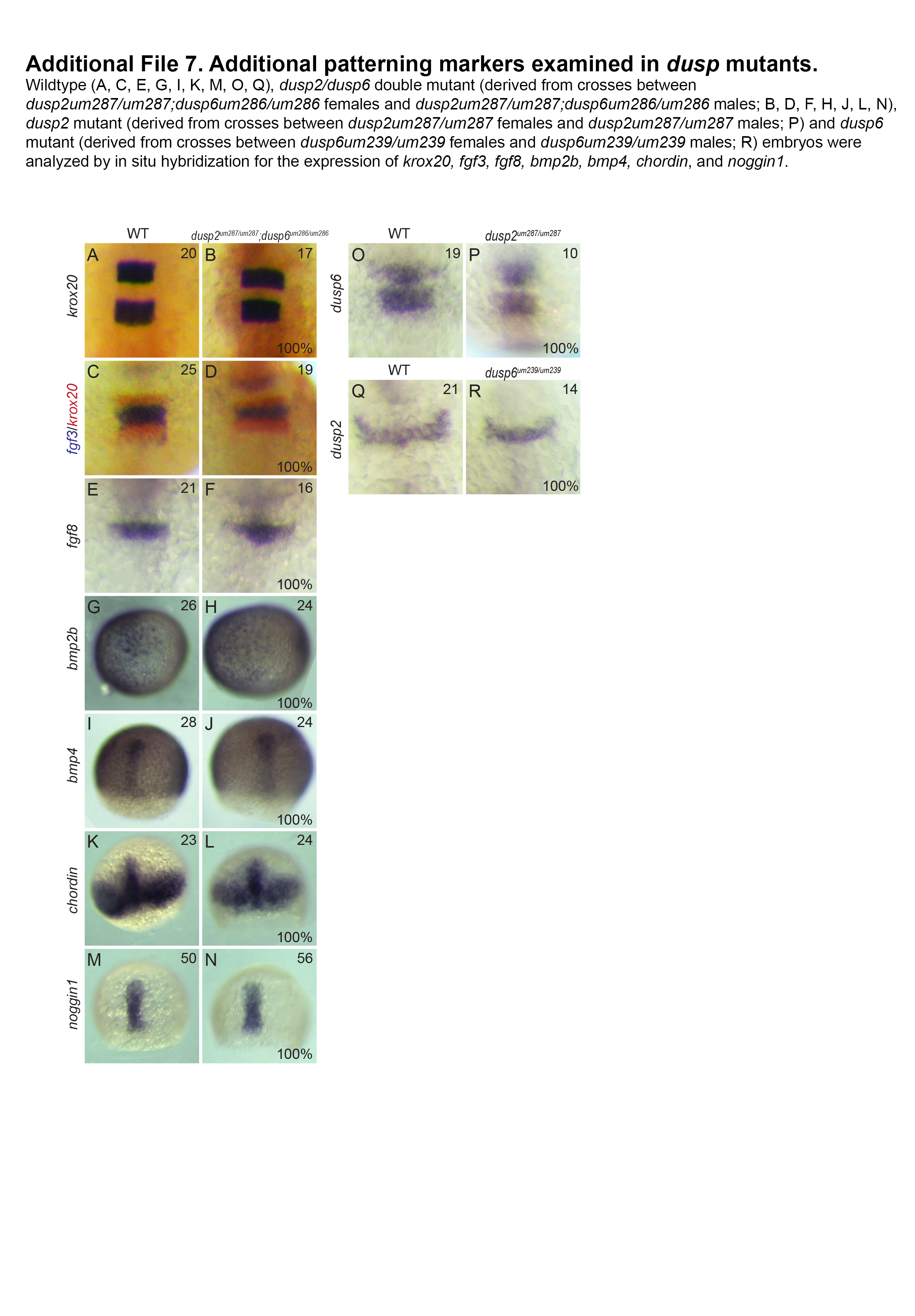

Supplement: Supplementary file 7 — Additional patterning markers examined in dusp mutants. Wildtype (A, C, E, G, I, K, M, O, Q), dusp2/dusp6 double mutant (derived from crosses between dusp2um287/um287;dusp6um286/um286 females and dusp2um287/um287;dusp6um286/um286 males; B, D, F, H, J, L, N), dusp2 mutant (derived from crosses between dusp2um287/um287 females and dusp2um287/um287 males; P) and dusp6 mutant (derived from crosses between dusp6um239/um239 females and dusp6um239/um239 males; R) embryos were analyzed by in situ hybridization for the expression of krox20, fgf3, fgf8, bmp2b, bmp4, chordin, and noggin1. (TIFF 3617 kb) [file 12861_2018_164_MOESM7_ESM.tif]

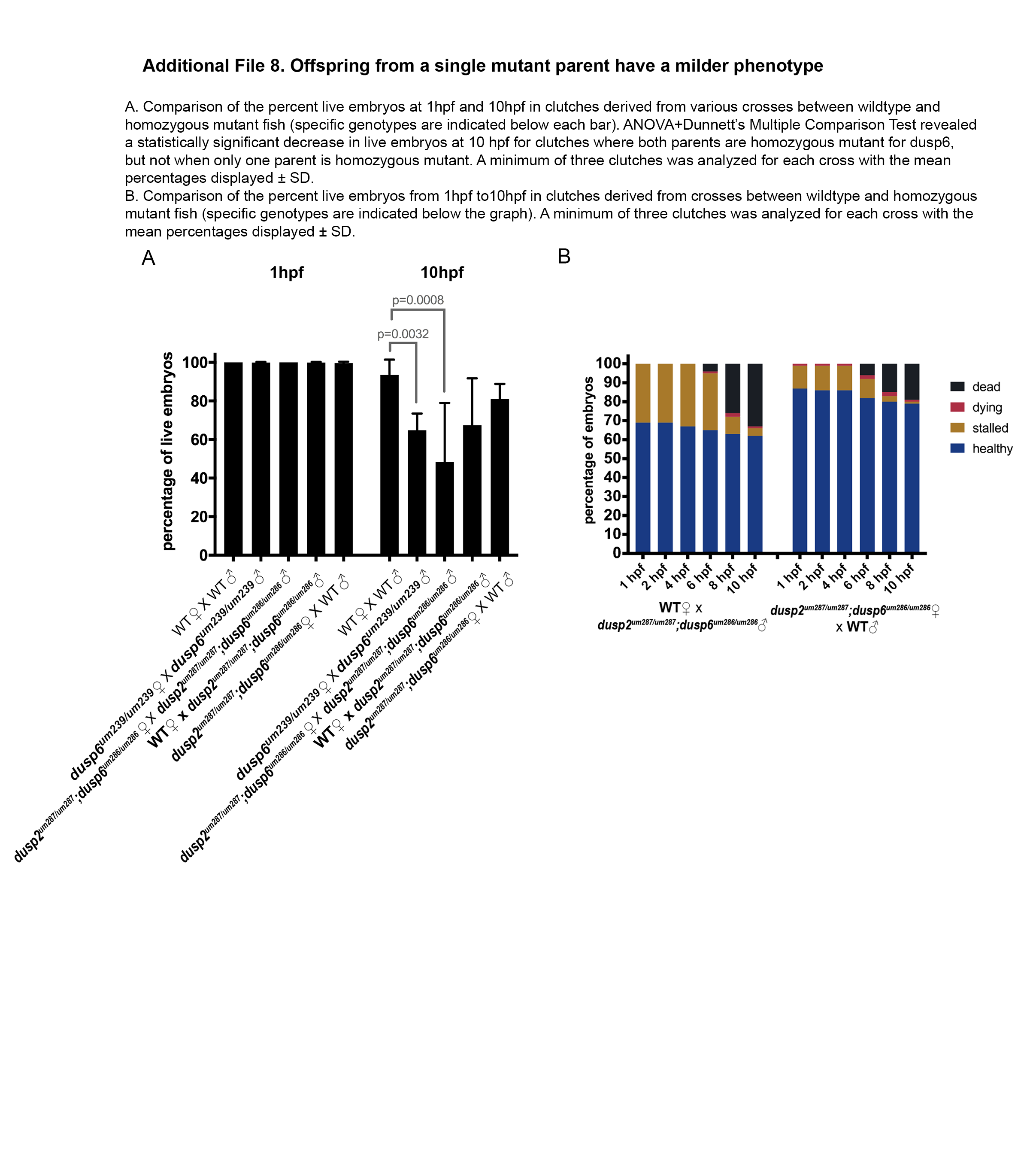

Supplement: Supplementary file 8 — Offspring from a single mutant parent have a milder phenotype. A. Comparison of the percent live embryos at 1hpf and 10hpf in clutches derived from various crosses between wildtype and homozygous mutant fish (specific genotypes are indicated below each bar). ANOVA+Dunnett’s Multiple Comparison Test revealed a statistically significant decrease in live embryos at 10 hpf for clutches where both parents are homozygous mutant for dusp6, but not when only one parent is homozygous mutant. A minimum of three clutches was analyzed for each cross with the mean percentages displayed ± SD. B. Comparison of the percent live embryos from 1hpf to10hpf in clutches derived from crosses between wildtype and homozygous mutant fish (specific genotypes are indicated below the graph). A minimum of three clutches was analyzed for each cross with the mean percentages displayed ± SD. (TIFF 2486 kb) [file 12861_2018_164_MOESM8_ESM.tif]

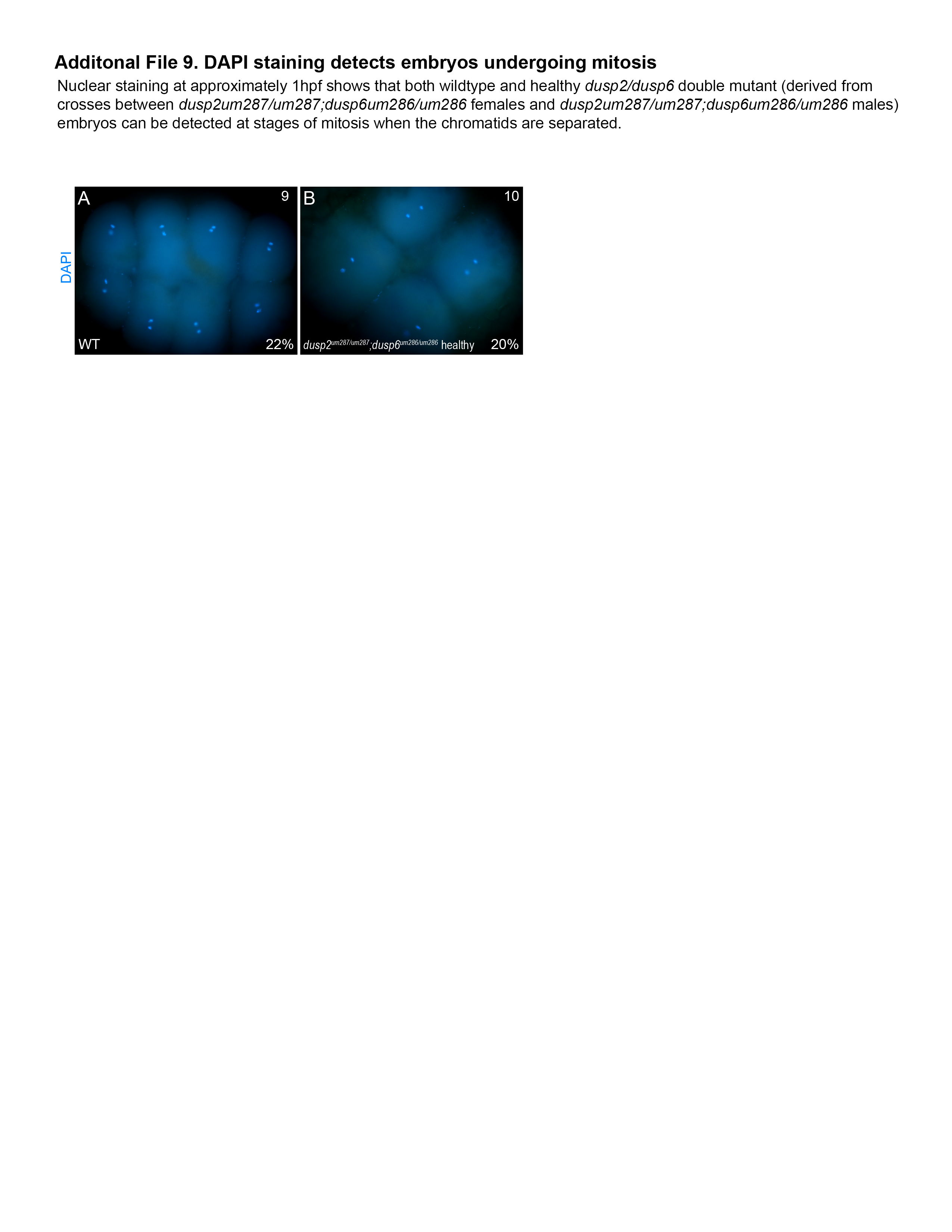

Supplement: Supplementary file 9 — DAPI staining detects embryos undergoing mitosis. Nuclear staining at approximately 1hpf shows that both wildtype and healthy dusp2/dusp6 double mutant (derived from crosses between dusp2um287/um287;dusp6um286/um286 females and dusp2um287/um287;dusp6um286/um286 males) embryos can be detected at stages of mitosis when the chromatids are separated. (TIFF 841 kb) [file 12861_2018_164_MOESM9_ESM.tif]
