# Supplementary material for: A parental requirement for dual-specificity phosphatase 6 in zebrafish
Source: BMC Dev Biol. 2018 Mar 15;18:6. doi: 10.1186/s12861-018-0164-6 (PMC5856328; doi:10.1186/s12861-018-0164-6)
Supplement: Supplementary file 6 — Differentially-expressed genes in the same body structures as dusp6 and dusp2. The left column contains a list of all body structures of the zebrafish in which dusp6 and/or dusp2 are expressed, and the right column contains the identified differentially-expressed genes also expressed in those structures. (DOCX 14 kb) [file 12861_2018_164_MOESM6_ESM.docx]

**Additional File 6. Differentially-expressed genes in the same body structures as *dusp6* and *dusp2***

| **Regions of the fish where *dusp6* and/or *dusp2* are expressed ^a^** | **Differentially-expressed genes also found in those regions ^b^** |
| --- | --- |
| axial hypoblast | *gsc* |
| blastodisc | *nanos3* |
|  | *gsc* |
| brain | *gria2a* |
|  | *nupr1* |
|  | *slc14a2* |
|  | *def6a* |
|  | *ca15b* |
|  | *irf8* |
|  | *cabp1a* |
|  | *prkcq* |
|  | *sult6b1* |
|  | *s100z* |
|  | *kiss1rb* |
|  | *tmie* |
|  | *rbp7a* |
|  | *kiss1* |
|  | *duox2* |
|  | *ptger3* |
|  | *dbh* |
| diencephalon | *kiss1rb* |
|  | *kiss1* |
|  | *gsc* |
| forebrain | *dock8* |
|  | *pth2r* |
| hindbrain | *grid2* |
|  | *kiss1rb* |
|  | *kiss1* |
|  | *sst1.2* |
|  | *dbh* |
|  | *pth2r* |
| hindbrain neural plate | *si:ch211-152c2.3* |
| hypothalamus | *gria2a* |
|  | *mchr1b* |
|  | *kiss1rb* |
|  | *sst1.2* |
| margin | *egln3* |
|  | *im:7138239* |
|  | *mespab* |
|  | *gsc* |
| midbrain | *pck1* |
|  | *dock8* |
|  | *kiss1rb* |
|  | *kiss1* |
|  | *pth2r* |
| mucus secreting cell | *zgc:92066* |
|  | *cabp1a* |
| neuron | *dbh* |
| optic vesicle | *pth2r* |
| otic vesicle | *si:ch211-152c2.3* |
|  | *zgc:92066* |
|  | *cabp1a* |
|  | *tmie* |
|  | *agbl4* |
|  | *gsc* |
|  | *pth2r* |
| pectoral fin | *gpib* |
|  | *def6a* |
|  | *ptgr1* |
|  | *mxra8a* |
|  | *gsc* |
| pectoral fin bud | *gsc* |
| peripheral olfactory organ | *zgc:92066* |
|  | *sult6b1* |
|  | *s100z* |
|  | *agbl4* |
| pharyngeal arch | *gpib* |
|  | *def6a* |
|  | *gsc* |
|  | *pth2r* |
| presumptive telencephalon | *gsc* |
| regenerating fin | *lcp1* |
| retina | *pck1* |
|  | *sh3bgrl2* |
|  | *ptgr1* |
|  | *six7* |
|  | *pth2r* |
| segmental plate | *zgc:92066* |
|  | *im:7138239* |
|  | *mespab* |
| shield | *gsc* |
| somite | *acta2* |
|  | *cpa4* |
|  | *zgc:92066* |
|  | *mxra8a* |
|  | *mespab* |
| tail bud | *szl* |
|  | *sult6b1* |
|  | *abcc6a* |
| telencephalon | *gria2a* |
|  | *grid2* |
|  | *sult6b1* |
|  | *kiss1rb* |
|  | *kiss1* |
|  | *sst1.2* |
|  | *gsc* |
| trunk | *bco2l* |
|  | *lcp1* |

^a^ Structures of the zebrafish in which *dusp6* and/or *dusp2* is expressed

^b^ Genes identified by RNA-seq that are expressed in those regions
